# Hormonal contraceptive use and depressive symptoms: systematic review and network meta-analysis of randomised trials

**DOI:** 10.1192/bjo.2021.64

**Published:** 2021-06-08

**Authors:** Anouk E. de Wit, Ymkje Anna de Vries, Marrit K. de Boer, Celeste Scheper, Ante A. Fokkema, Robert A. Schoevers, Erik J. Giltay

**Affiliations:** Department of Psychiatry, University of Groningen, University Medical Center Groningen, The Netherlands; Department of Developmental Psychology, University of Groningen, The Netherlands; Department of Psychiatry, University of Groningen, University Medical Center Groningen, The Netherlands; Department of Psychiatry, University of Groningen, University Medical Center Groningen, The Netherlands; Department of Psychiatry, University of Groningen, University Medical Center Groningen, The Netherlands; Department of Psychiatry, University of Groningen, University Medical Center Groningen, The Netherlands; Department of Psychiatry, University Medical Center Leiden, The Netherlands

**Keywords:** Randomised controlled trial, depressive disorders, epidemiology, neuroendocrinology, outcome studies

## Abstract

**Background:**

Observational studies suggest that hormonal contraceptive use may increase depressive symptoms in women, but it is unclear whether the effect is causal.

**Aims:**

To quantitatively examine the evidence from randomised clinical trials for the link between hormonal contraceptive use and depressive symptoms.

**Method:**

We performed a systematic review and network meta-analysis of randomised clinical trials comparing women randomised to any form of a hormonal contraceptive with women randomised to any other form of a (non-)hormonal contraceptive or placebo. We searched the Cochrane Central Register of Controlled Trials (CENTRAL), PubMed, Web of Science, PsycINFO, EMCare and EMBASE, from inception to 1 May 2020. Certainty of the evidence was assessed with the Grading of Recommendations Assessment, Development and Evaluation approach. A random-effect Bayesian network meta-analysis was conducted, with change in depressive symptoms between baseline and three cycles as outcome.

**Results:**

This review identified 3492 records, of which 14 trials were eligible and 12 could be included in the network meta-analysis. These trials included 5833 participants (mean age per study range: 16.8–32.4 years) and compared 10 different interventions. Compared with placebo, hormonal contraceptive use did not cause worsening of depressive symptoms (standardised mean difference: median, −0.04; range, −0.17 [95% credible interval −0.46 to 0.13] to 0.13 [95% credible interval −0.28 to 0.56]).

**Conclusions:**

This study suggests that hormonal contraceptive use does not lead to an increase in depressive symptoms in adult women. Future studies should include first-time users, to confirm the results in young women.

There has been considerable debate whether hormonal contraceptives influence affective states. Although cohort studies have suggested that hormonal contraceptive use could increase the risk of depressive symptoms or the prescription of psychotropic drugs,^1–4^ the use of observational data precludes statements about the causality of this relationship. A meta-analysis of experimental data could confirm or reject the causality of this observation, but no such meta-analysis has been performed to date.^5,6^

## Use of hormonal contraceptives and depressive symptoms

The execution of such an analysis is complicated by the availability of numerous hormonal contraceptives that each may have different effects.^5^ The best-known subgroup of hormonal contraceptives is the combined oral contraceptive pill (COC), which contains synthetic forms of oestrogen and progestin. Other examples of hormonal contraceptives are hormonal intrauterine devices and progestin-only implants (or pills of injections) that only contain a synthetic progestin. Some hormonal contraceptives have been suggested as possessing a higher risk of depressive symptoms compared with other contraceptives, including those containing higher doses of synthetic oestrogens^2,7^ and those containing progestins with androgenic features.^8,9^ Cyclic versus continuous regimens may also increase the risk of developing depressive symptoms.^10^ Additionally, large observational studies have shown that teenage girls who used hormonal contraceptives in particular reported more depressive symptoms than those who did not use hormonal contraceptives,^1,2,4^ which might suggest that younger women are more vulnerable to the adverse effects of hormonal contraceptives independent of the specific formulation used. However, it may also point to the ‘healthy user effect’, which occurs when women who start hormonal contraceptive use as a teenager and experience side-effects are more likely to discontinue use.^11^ If true, the stronger effect in younger women is explained by the higher proportion of first-time users, but not by younger age itself.

## Aims of this systematic review and network meta-analysis

Given the substantial disease burden of depression, and the importance of hormonal contraceptives for the prevention of unwanted pregnancies and the treatment of dysmenorrhea and acne, it is essential to examine whether hormonal contraceptives may cause depressive symptoms. A network meta-analysis of randomised clinical trials (RCTs) makes it possible to estimate the comparative harm of different hormonal contraceptives, using both direct and indirect evidence, and hence to summarise and interpret the evidence for their putative effect on depressive symptoms.^12^ Here, we conducted a systematic review and network meta-analysis on data from RCTs that assessed the effects of hormonal contraceptives, compared with any other (non-)hormonal contraceptive regimen, on depressive symptoms in women. Our primary aim was to examine whether any hormonal contraceptive compared with each other or placebo, COCs with higher versus those with lower doses of oestrogen, hormonal contraceptives with androgenic progestins versus those with anti-androgenic progestins or COCs with a cyclic regimen versus those with a continuous regimen, had a more negative effect on depressive symptoms. Our secondary aim was to investigate whether younger women are especially at risk for such potential side-effects.

## Method

### Search strategy and selection criteria

RCTs were eligible for inclusion if they included premenopausal women, compared hormonal contraceptive regimens with each other or with non-hormonal contraceptives (like placebo or copper intrauterine device) and evaluated depressive symptoms with a validated (self-report or observer-rated) scale. Hormonal contraceptives included COCs, combined injectable contraceptives, contraceptive patches, contraceptive rings, progestin-only pills, progestin-only injectables, progestin-only implants and levonorgestrel-releasing intrauterine devices. Studies performed in transwomen, women with an oophorectomy or women with hypogonadism were excluded. Studies that investigated hormonal contraceptives as a treatment for premenstrual dysphoric disorder or syndrome were also excluded because they target mood as a therapeutic goal and not as an unintended side-effect.

We conducted a search for English-language papers in the Cochrane Central Register of Controlled Trials, PubMed, Web of Science, PsycINFO, EMCare and EMBASE, from their inception to 1 May 2020. Reference lists of included trials were checked to identify other potentially eligible trials or ancillary publications. Roughly, we used multiple search terms to find ‘randomised clinical trials’ that studied any form of ‘hormonal contraceptive’ and included assessments that measured ‘depressive symptoms or depression’. See Supplementary Appendix 1 available at https://doi.org/10.1192/bjo.2021.64 for the full electronic searches.

Two reviewers (C.S. and A.A.F.) independently scanned all retrieved citations by title, abstract and full text, according to the prespecified inclusion criteria. Any discrepancies were resolved through discussion or recourse to a third reviewer (A.E.d.W.). Two reviewers (C.S. and A.A.F.) extracted data on summary estimates independently for each eligible trial, using a standardised pilot-tested data extraction form. The first data were extracted on 6 July 2020. This study is registered with the International Prospective Register of Systematic Reviews (PROSPERO; identifier CRD42020193304).

### Data analysis

The reviewers (C.S. and A.A.F.) independently collected information on methodology (level of blinding, cross-over or parallel-group design), interventions (formulation, dose, frequency, regimen/route of administration), participants (participants per group, number who dropped out, age, first-time use of hormonal contraceptives, sexual activity, comorbidity) and outcomes (tools/scales, time points reported, phase of cycle reported). Outcomes included change in depressive symptoms or onset of a new depression between baseline and three cycles. The cycle represented the length of one completed treatment period as it was defined by the original research, and therefore the duration of the cycle may slightly differ between trials. For example, some trials used a 24-day active and 4-day hormone-free interval, whereas others used a 21-day active and 7-day hormone-free interval. When data for three cycles were not available, other data as close to this point as possible were used (eligible range of 1–48 cycles). Preference was given to validated depressive symptom questionnaires, but when such questionnaires were not used in a trial, data from negative affect or depressive symptom subscales from scales measuring related concepts were accepted. When depressive symptoms or depression were measured with more than one standardised rating scale, the scale with the best psychometric properties (according to validity, reliability, responsiveness and interpretation) was chosen. However, in practice, we did not have to choose between scales because none of the trials reported useful data for more than one scale. If results were reported separately for different cycle phases, effect sizes were averaged across phases. Intention-to-treat data were used whenever possible. Reviewers resolved discrepancies by discussion and, when necessary, adjunction by a third party (A.E.d.W.). We contacted study authors and drug manufactures to supplement incomplete reports of the original papers. The relative effect per comparison was summarised with the standardised mean difference (SMD), adjusted for small sample sizes (Hedges’ *g* correction), with a 95% credible interval.^13^

We performed a random-effects Bayesian network meta-analysis to explore all direct and indirect comparisons, using the ‘*gemtc*’ package in R version 4.0.3 for Mac OS X (R Foundation for Statistical Computing, Vienna, Austria; see https://cran.r-project.org/bin/macosx/). Trials that were not connected to the network were excluded from the network meta-analysis, and we only reported the direct effects for such comparisons. We accounted for the correlations induced by multi-arm studies by using multivariate distributions. We used uninformative, default priors for model parameters, and ran Markov chain Monte Carlo sampling. Simulations were run for four chains with an adaptive phase of 10 000 and a sampling phase of 500 000 iterations, thinned such that every tenth iteration was retained. The convergence of the models was checked by trace plots, density plots and the Brooks–Gelman–Rubin diagnostic. Each type of hormonal contraceptive (including regimen and dose) was treated as a separate node. A network plot was drawn, with thickness of the lines between nodes based on the number of direct comparisons investigated.

To facilitate interpretation of the findings, we calculated the median SMDs of all hormonal contraceptives and presented the range of SMDs of individual formulations. Then, we calculated the median SMD of the following groups: COCs with higher versus those with lower doses of oestrogen, hormonal contraceptives with androgenic progestins versus those with anti-androgenic progestins and COCs with a cyclic regimen versus those with a continuous regimen. Finally, we used a meta-regression analysis to examine whether studies that included on average younger women reported larger effect sizes, compared with placebo, than those that included older women. The mean age of participants in the studies (in years) was standardised. The beta coefficient and 95% credible interval of age, together with the change in deviance information criterion (DIC), was used to determine the effect of age on the relationship between hormonal contraceptive use and depressive symptoms.

The overall certainty of evidence was determined with the Confidence in Network Meta-analysis 1.9.1 (CINeMA, Evidence Synthesis Methods group, ISPM unibe.ch; see https://cinema.ispm.unibe.ch) online tool, based on the Grading of Recommendations Assessment, Development and Evaluation (GRADE) for network meta-analysis.^14–16^ Judgements on the certainty of evidence were made for each of the following domains: within-study bias, reporting bias, indirectness, imprecision, heterogeneity and incoherence. The risk of within study biases for the outcome measure depressive symptoms was assessed with the modified Cochrane Risk of Bias tool 2.0.^17^ For full details on the GRADE assessment, see Supplementary Appendix 2.

## Results

**Fig. 1:**
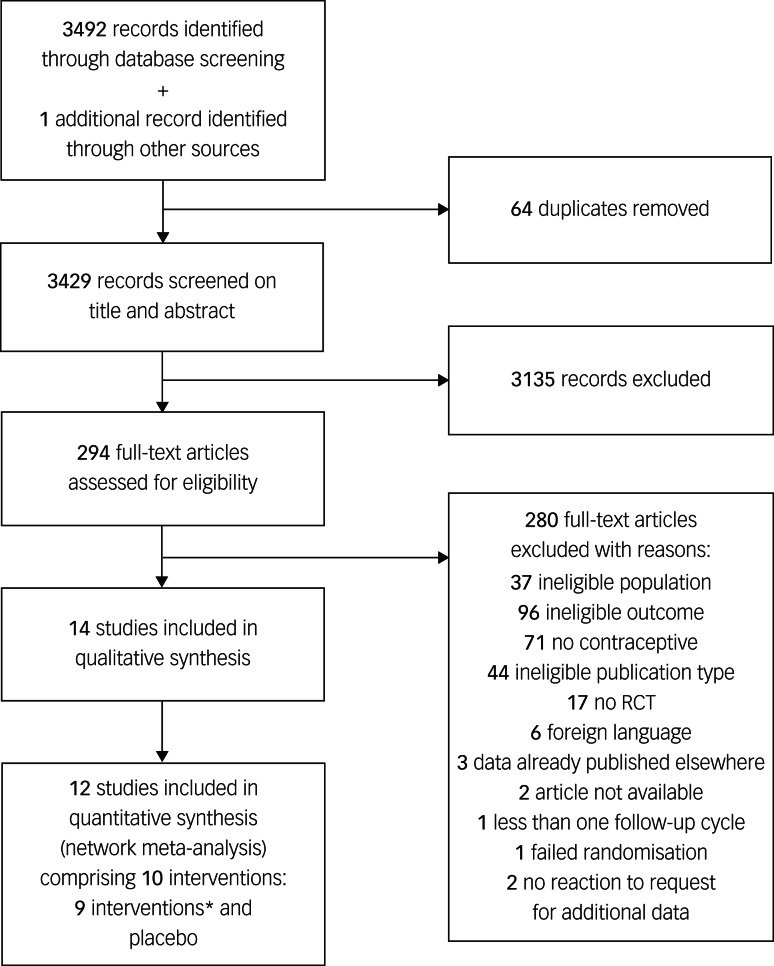
Preferred Reporting Items for Systematic Reviews and Meta-Analyses flow diagram. *Ethinylestradiol/drospirenone (30 *μ*g/3 mg), ethinylestradiol/etonogestrel (15 *μ*g/120 *μ*g), oestradiol/dienogest (3;2;2;1 mg/0;2;3;0 mg), desogestrel (75 *μ*g), ethinylestradiol/levonorgestrel (30 *μ*g/150 *μ*g), levonorgestrel (30 *μ*g), oestradiol/nomegestrol acetate (1.5 mg/2.5 mg), ethinylestradiol/levonorgestrel (20 *μ*g/100 *μ*g) and ethinylestradiol/desogestrel (20 *μ*g/150 *μ*g). RCT, randomised clinical trial.

Our systematic search identified 3492 citations published between 1961 and 2020, of which 15 articles were considered eligible (Fig. 1).^7–9,18–29^ One additional trial was identified by hand-searching other articles.^30^ The information available in five of these articles was not sufficient to complete the data collection,^18–20,26,31^ but authors from three trials provided us with the missing data.^19,20,26^ One study was excluded after data extraction, as the baseline depressive symptom scores differed by >5 s.d. between treatment groups, suggesting a failure of randomisation and rendering the results uninterpretable.^29^ Out of the remaining 14 studies, two had no connection with the network,^7,30^ and are therefore only qualitatively described in this review.

Of the 12 trials included in the network meta-analysis, 11 had a parallel design and 1 had a cross-over design.^8,9,18–27^ A total of ten different interventions were examined (nine active and one placebo). Six active interventions were COCs, two were progesterone-only pills and one was a vaginal ring. Two multi-arm trials compared three interventions, of which one additionally included two separate samples.^19,20^ The remaining were two-arm trials.^8,9,18,21–27^ A total of 5833 women were included, but across comparisons, the sample size per arm ranged from 14 to 2631 participants (Table 1). The weighted average duration of follow-up was 3.6 cycles (range 1–6), and the weighted mean drop-out rate was 16.6% (range 1.3–24.2%). Women had a weighted mean age of 27.3 years (range 16.8–32.4), and were, if studies provided such information, often sexually active (weighted mean rate 63.2%).^19,20,22,23,25–27^ Information on previous hormonal contraceptive use was provided in only five studies. Of these studies, two did not include first-time users at all,^24,26^ and the others included between 1.3 and 33.0% of first-time users.^20,21,23^ Most studies excluded women with a current depression or antidepressant use,^20,23,24,26,27^ or did not provide information on baseline depression presence.^8,9,19,22,28^ Only a few studies specifically included women with current depression^21,25^ or previous hormonal contraceptive-related negative affect,^21,24^ to make the sample as broadly representative as possible.

**Table 1:** Characteristics of the included randomised controlled trials

	Age, years	Intervention		Comparison		First-time users,	Number who dropped out	Duration	RCT type	Analysis	Depression scale
	Mean (s.d.)	Formulation	*n*^a^	Formulation	*n*^a^	%^b^	*n* (%)	Number of cycles			
Battaglia et al^27^	26.5 (2.4)	Ethinylestradiol/drospirenone (30 *μ*g/3 mg)	22	Ethinylestradiol/etonogestrel (15 *μ*g/120 μg)	21	?	3 (7.0)	6	Parallel, single blinded	Complete cases	BDI
Davis et al^26^	30.5 (7.4)	Oestradiol/dienogest (3;2;2;1 mg/0;2;3;0 mg)	97	Ethinylestradiol/levonorgestrel (30 *μ*g/150 *μ*g)	103	0.0	13 (6.1)	4	Parallel, double blinded	Complete cases	PGWBI Depression
Elaut et al^19^	23.1 (4.3)	Ethinylestradiol/etonogestrel (15 *μ*g/120 mg), desogestrel (75 *μ*g)	55 55	Ethinylestradiol/desogestrel (20 *μ*g/150 *μ*g)	55	?	40 (24.2)	3	Cross-over, unblinded	Intention to treat	SCL-90 Depression
Engman et al^24^	24.9 (4.2)	Ethinylestradiol/levonorgestrel (30 *μ*g/150 *μ*g)	14	Placebo	15	0.0	4 (11.4)	1	Parallel, double blinded	Complete cases	MADRS
Graham et al, sample a^20^	32.4 (3.7)	Ethinylestradiol/levonorgestrel (30 *μ*g/150 *μ*g), levonorgestrel (30 *μ*g)	24 22	Placebo	25	1.3	4 (5.3)	3	Parallel, double blinded	Complete cases	BDI
Graham et al, sample b^20^	32.2 (4.2)	Ethinylestradiol/levonorgestrel (30 *μ*g/150 *μ*g), levonorgestrel (30 μg)	24 25	Placebo	25	33.0	1 (1.3)	3	Parallel, double blinded	Complete cases	BDI
Greco et al^7^	19.8 (1.8)	Ethinylestradiol/norgestimate (35 *μ*g/0.18;0.22;0.25 mg)	31	Ethinylestradiol/norgestimate (25 *μ*g/0.18;0.22;0.25 mg)	29	?	12 (20.0)	3	Parallel, single blinded	Complete cases	BDI
Kelly et al^8^	26.5 (6.1)	Ethinylestradiol/drospirenone (30 *μ*g/3 mg)	193	Ethinylestradiol/levonorgestrel (30 *μ*g/150 *μ*g)	87	?	144 (34.0)	6	Parallel, single blinded	Complete cases	MDQ Negative
Legro et al^30^	27.2 (5.1)	Ethinylestradiol/norethindrone acetate (20 *μ*g/1 mg continuous)	31	Ethinylestradiol/norethindrone acetate (20 *μ*g/1 mg)	31	?	11 (17.7)	6	Parallel, double blinded	Intention to treat	MDQ Negative
Lundin et al^21^	24.3 (4.2)	Oestradiol/nomegestrol acetate (1.5 mg/2.5 mg)	80	Placebo	88	17.8	24 (11.9)	3	Parallel, double blinded	Complete cases	MADRS
O'Connell et al^28^	16.8 (2.1)	Ethinylestradiol/levonorgestrel (20 *μ*g/100 *μ*g)	34	Placebo	34	?	8 (10.5)	3	Parallel, double blinded	Complete cases	CES-D
Sangthawan and Taneepanichskul^9^	26.8 (4.5)	Ethinylestradiol/drospirenone (30 *μ*g/3 mg)	50	Ethinylestradiol/levonorgestrel (30 *μ*g/150 *μ*g)	49	?	5 (4.8)	6	Parallel, unblinded	Complete cases	WHAQ Negative
Winkler et al^23^	28.3 (?)	Ethinylestradiol/desogestrel (20 *μ*g/150 *μ*g)	404	Ethinylestradiol/levonorgestrel (20 *μ*g/100 *μ*g)	384	7.6	239 (23.3)	6	Parallel, unblinded	Complete cases	PGWBI Depression
Witjes et al^22^	27.6 (7.0)	Oestradiol/nomegestrol acetate (1.5 mg/2.5 mg)	2631	Ethinylestradiol/drospirenone (30 *μ*g/3 mg)	891	?	?	3	Parallel, unblinded	Intention-to-treat	MDQ
Zethraeus et al^25^	23.7 (3.6)	Ethinylestradiol/levonorgestrel (30 *μ*g/150 *μ*g)	162	Placebo	167	?	11 (3.2)	3	Parallel, double blinded	Complete cases	BDI

RCT, randomised clinical trial; BDI, Beck Depression Inventory; CES-D, Center for Epidemiologic Studies Depression Scale; MADRS, Montgomery-Äsberg Depression Rating Scale; MDQ neg, Menstrual Distress Questionnaire negative affect subscale; PGWBI dep, Psychological General Well-Being Index depression subscale; SCL-90 dep, Symptom Checklist 90 depression subscale; WHAQ neg, Women's Health Assessment Questionnaire negative affect subscale. Ethinylestradiol/dienogest (3;2;2;1 mg/0;2;3;0 mg) is a multiphasic combined oral contraceptive that has four different dosages of hormones throughout a 4-week cycle. The dosages before the ‘slash’ refer to the different dosages of ethinylestradiol, and the ones after the ‘slash’ refer to those of dienogest. The study by Graham et al included two different samples, of which sample a was from the Philippines and sample b was from Scotland.

a. Number of women who were available for the analysis (excluding those who dropped out) in case of complete-case analyses.

b. Percentage of the baseline sample that had no experience with hormonal contraceptive use before participating in the trial. As the majority of studies ran complete-case analyses, it is unknown what percentage of women included in the meta-analyses had previous hormonal contraceptive experience.

**Fig. 2:**
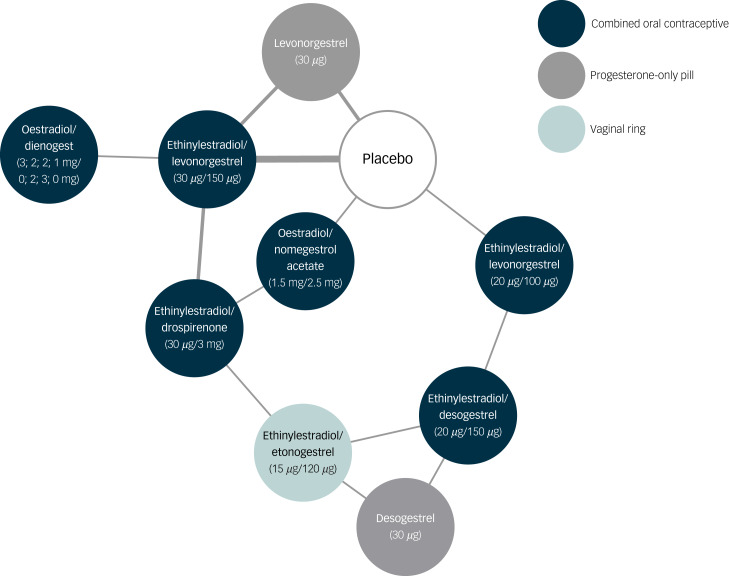
Network of treatment comparisons for depressive symptoms, including forest plot with results. Each node represents different a different hormonal contraceptive or placebo. The thickness of lines between nodes is proportional to the number of studies investigating the direct comparison.

**Fig. 3:**
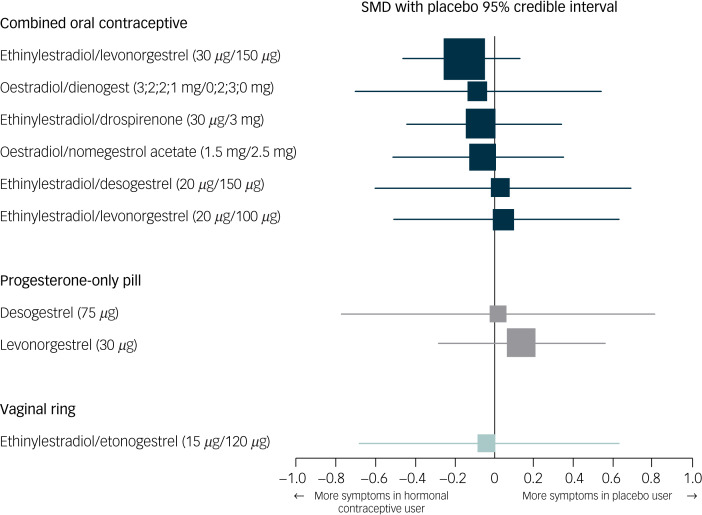
Network forest plot of results compared with placebo. The size of the SMD dots are proportional to the precision of the estimate (one per s.e.). Ethinylestradiol/dienogest (3;2;2;1 mg/0;2;3;0 mg) is a multiphasic combined oral contraceptive that has four different dosages of hormones throughout a 4-week cycle. The dosages before the ‘slash’ refer to the different dosages of ethinylestradiol, and the ones after the ‘slash’ refer to those of dienogest. SMD, standardised mean difference.

The network of treatment comparisons included ten individual nodes (Fig. 2). Placebo and ethinylestradiol/levonorgestrel (30 *μ*g/150 *μ*g) were the most well-connected interventions, with eight other interventions directly linked to each. The network meta-analysis showed that none of the hormonal contraceptives worsened depressive symptoms compared with placebo (median SMD −0.04, range SMD −0.17 [95% credible interval −0.46 to 0.13] to 0.13 [95% credible interval −0.28 to 0.56]) (Fig. 3).

**Fig. 4:**
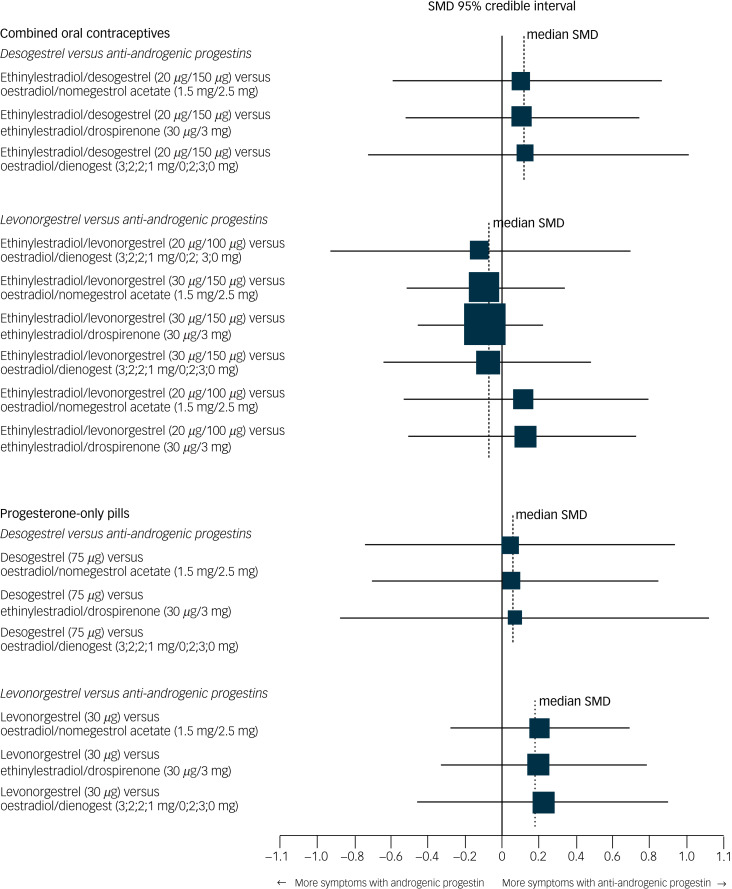
Network forest plot of results of hormonal contraceptives with an androgenic progestin versus those with an anti-androgenic progestin. Two androgenic progestins included in the network (levonorgestrel and desogestrel) were compared with the anti-androgenic progestins included in the network (dienogest, drospirenone, nomegestrol acetate). Synthetic progestins, especially the older ones, not only have affinity to the progesterone receptor, but can also have androgenic or oestrogenic actions. From the synthetic progestins here, levonorgestrel has the strongest affinity to the androgen receptor, followed by desogestrel.^32^ Newer progestins have higher progesterone potency and additional effects, such as anti-androgenic activity.^32^ The size of the SMD dots are proportional to the precision of the estimate (one per s.e.). Ethinylestradiol/dienogest (3;2;2;1 mg/0;2;3;0 mg) is a multiphasic combined oral contraceptive that has four different dosages of hormones throughout a 4-week cycle. The dosages before the ‘slash’ refer to the different dosages of ethinylestradiol, and the ones after the ‘slash’ refer to those of dienogest. SMD, standardised mean difference.

**Fig. 5:**
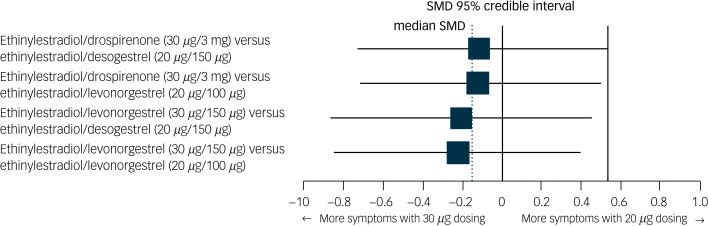
Network forest plot of results of combined oral contraceptives with 30 *μ*g ethinylestradiol versus those with 20 *μ*g ethinylestradiol. Two doses of ethinylestradiol were included in network: 20 mg and 30 mg). The size of the SMD dots are proportional to the precision of the estimate (one per s.e.). SMD, standardised mean difference.

None of the specific formulations of hormonal contraceptives had a significantly more negative effect on depressive symptoms than any other specific formulation, suggesting that there was no group of hormonal contraceptives causing more depressive symptoms than another group. See Table 2 for all of the comparisons of effects of hormonal contraceptives on depressive symptoms. First, hormonal contraceptives with androgenic progestins (e.g. levonorgestrel and desogestrel) did not have a stronger association with depressive symptoms than those with anti-androgenic progestins (i.e. dienogest, nomegestrol acetate or drospirenone). For example, the median SMDs for COCs containing levonorgestrel or desogestrel compared with those containing anti-androgenic progestins were −0.09 and 0.11, respectively (Fig. 4). Second, COCs with higher doses of oestrogen did not induce more depressive symptoms compared with those with lower doses (Fig. 5; median SMD of −0.16 for formulations with 30 *μ*g compared with 20 *μ*g ethinylestradiol). This was also supported by a study that lacked a connection with the network.^7^ In that study, women who used a COC with a lower dose of oestrogen (ethinylestradiol/norgestimate, 25 *μ*g/180 *μ*g/215 *μ*g/250 *μ*g) had on average fewer depressive symptoms than women using the same contraceptive with a higher dose of oestrogen (35 *μ*g/180 *μ*g), but this was not statistically significant (mean difference in Beck Depression Inventory score between low- and high-oestrogen group, −2.26; *P* = 0.09). As the doses of oestradiol and ethinylestradiol are hard to compare and one of the two studies that studied oestradiol used a COC with a four-phasic regimen, which makes it even harder to estimate the average level of oestradiol to use, we limited our analyses to the direct and indirect effects of COCs including ethinylestradiol. Third, we aimed to compare continuous and intermittent regimens, but no such comparison was included in the network. However, another study that could not be included in the network showed that women who used ethinylestradiol/norgestimate (20 *μ*g/1 mg) continuously did not have fewer depressive symptoms after 6 months of use than those who took it cyclically (mean difference in negative affect on Moos Menstrual Distress Premenstrual Questionnaire *T*-scores, −2.8; *P* = 0.32).^30^

**Table 2:** Comparisons for effects of hormonal contraceptives on depressive symptoms

Desogestrel (75 *μ*g)									
0.09^‡^ (−0.88 to 1.07)	Oestradiol/dienogest (3;2;2;1 mg/0;2;3;0 mg)								
0.06^‡^ (−0.75 to 0.94)	−0.04^‡^ (−0.71 to 0.72)	Oestradiol/nomegestrol acetate (1.5 mg/2.5 mg)							
0.04^‡^ (−0.54 to 0.62)	−0.05^‡^ (−0.91 to 0.80)	−0.02^‡^ (−0.76 to 0.66)	Ethinylestradiol/etonogestrel (15 *μ*g/120 *μ*g)						
−0.03^‡^ (−0.61 to 0.55)	−0.13^‡^ (−1.00 to 0.74)	−0.10^‡^ (−0.85 to 0.60)	−0.08^‡^ (−0.61 to 0.46)	Ethinylestradiol/desogestrel (20 *μ*g/150 *μ*g)					
−0.04^‡^ (−0.74 to 0.66)	−0.14^‡^ (−0.96 to 0.69)	−0.11^‡^ (−0.81 to 0.55)	−0.09^‡^ (−0.71 to 0.55)	−0.01^‡^ (−0.47 to 0.46)	Ethinylestradiol/levonorgestrel (20 *μ*g/100 *μ*g)				
0.07^‡^ (−0.71 to 0.85)	−0.02^‡^ (−0.68 to 0.62)	0.03^‡^ (−0.44 to 0.37)	0.03^‡^ (−0.59 to 0.64)	0.11^‡^ (−0.56 to 0.76)	0.12^‡^ (−0.52 to 0.74)	Ethinylestradiol/drospirenone (30 *μ*g/3 mg)			
0.18^‡^ (−0.62 to 0.98)	0.08^‡^ (−0.47 to 0.63)	0.12^‡^ (−0.37 to 0.53)	0.13^‡^ (−0.52 to 0.80)	0.21^‡^ (−0.46 to 0.88)	0.22^‡^ (−0.40 to 0.84)	0.10^‡^ (−0.22 to 0.45)	Ethinylestradiol/levonorgestrel (30 *μ*g/150 *μ*g)		
−0.12^‡^ (−1.00 to 0.74)	−0.22^‡^ (−0.91 to 0.46)	−0.18^‡^ (−0.79 to 0.35)	−0.17^‡^ (−0.92 to 0.58)	−0.09^‡^ (−0.85 to 0.65)	−0.08^‡^ (−0.79 to 0.61)	−0.19^‡^ (−0.70 to 0.31)	−0.30^‡^ (−0.71 to 0.10)	Levonorgestrel (30 *μ*g)	
0.01^‡^ (−0.77 to 0.81)	−0.09^‡^ (−0.70 to 0.54)	−0.05^‡^ (−0.51 to 0.35)	−0.04^‡^ (−0.68 to 0.63)	0.04^‡^ (−0.60 to 0.69)	0.05^‡^ (−0.52 to 0.63)	−0.07^‡^ (−0.44 to 0.34)	−0.17^‡^ (−0.46 to 0.13)	0.13^‡^ (−0.28 to 0.56)	Placebo

Data are standardised mean differences (SMD) with 95% credible intervals in the column-defining intervention compared with the row-defining intervention. Higher SMD values correspond with fewer depressive symptoms in the column-defining hormonal contraceptive. None of the results were significant and all evidence was of low certainty (marked with ^‡^). Ethinylestradiol/dienogest (3;2;2;1 mg/0;2;3;0 mg) is a multiphasic combined oral contraceptive that has four different dosages of hormones throughout a 4-week cycle. The dosages before the ‘slash’ refer to the different dosages of ethinylestradiol, and the ones after the ‘slash’ refer to those of dienogest.

The meta-regression analyses showed that on average, every year decrease in mean age of the women investigated in a trial was associated with 0.028 lower SMD of the hormonal contraceptive group compared with the placebo group (lower SMD indicating more depressive symptoms). However, this was not significant (standardised beta coefficient for age, 0.238; 95% credible interval −0.232 to 0.708; *Δ*DIC, −3.6). This association weakened after exclusion of the study that solely included women with previous hormonal contraceptive-related negative affect (beta coefficient for age, 0.193; 95% credible interval −0.254 to 0.642; *Δ*DIC, −4.0).^24^

The certainty of evidence (GRADE) for all comparisons was judged to be ‘very low’ (Supplementary Appendix 2 and Table 1). Most of these judgements were caused by within-study bias (Supplementary Appendix 2.1), and by indirectness (Supplementary Appendix 2.3) and imprecision (Supplementary Appendix 2.4) of the effect sizes. The judgements made for each of the domains are described in detail in Supplementary Appendix 2.

## Discussion

This systematic review examined 14 RCTs, of which 12 were included in a network meta-analysis to assess the effects of hormonal contraceptive use on depressive symptoms. Data from 5833 women were available, and 9 different hormonal contraceptives were compared with each other and placebo. The results suggest that, with a very low certainty of evidence, none of the investigated hormonal contraceptives caused more depressive symptoms, compared with placebo.

Here, we have provided quantitative evidence, based on RCTs, that hormonal contraceptives appear to have a limited effect on depressive symptoms. This finding is largely in line with the suggestions from previous qualitative reviews.^5,6,33,34^ In contrast to those reviews, however, we could not confirm the idea that the dose of ethinylestradiol or the type of progestin influences the effect of hormonal contraceptives on depressive symptoms. Although the data regarding the safety of 20 *μ*g *v*. 30 *μ*g ethinylestradiol COCs suggested less risk with 20 *μ*g formulas, this was not strong enough to endorse higher safety with 20 *μ*g pills. We cannot rule out that much higher doses of ethinylestradiol (e.g. 50 *μ*g) adversely affect the affective state, as none of the included trials studied such high doses, but these formulations are not recommended for contraception. Formulations containing androgenic progestins also did not cause more depressive symptoms than those with anti-androgenic formulations.

The suggestion that hormonal contraceptives have no effect on depressive symptoms stands in direct opposition to results from several observational studies showing that women who use hormonal contraceptives are at increased risk for depression or report more depressive symptoms.^1–4^ Residual confounding in observational studies could be one compelling explanation for the contradiction in findings. Indeed, one observational study showed that the association between COC use and depressive symptoms could at least be partly explained by pre-existing differences between women who use oral contraceptives and those who do not, which included differences in number of women being sexually active, number of stressful experiences and number of women having acne or menstrual related pain.^1^ However, the absence of a significant effect in the current meta-analysis may also have arisen because of limitations of the currently available randomised trials. As some observational studies showed that an increased prevalence of depressive symptoms related to hormonal contraceptives was most pronounced in teenage girls,^1–4^ this may suggest that the absence of an effect in the, on average, adult women in our study could be a result of the healthy user effect.^11^ We could not confirm that this bias has occurred in our analysis because effects of hormonal contraceptives on depressive symptoms were not stronger in studies that included on average younger women. However, age may be an imperfect proxy for first-time use, especially as there was only one trial that included teenage girls, whereas the majority of trials included women in their mid-20s. Hence, we cannot rule out that first-time use is a risk factor for experiencing depressive symptoms with hormonal contraceptive use.

Therefore, to provide a definite answer to the question of whether hormonal contraceptives adversely affect depressive symptoms, more studies are needed that also include first-time users, to ensure that findings are not biased by a healthy user effect. Women who have had experienced side-effects of hormonal contraceptives during their first-time use are less likely to consent to participate in a randomised trial of hormonal contraceptives, making it unlikely that these side-effects are going to be observed. As the potential randomisation to a less reliable non-hormonal contraceptive, such as a condom, would likely be ethically unacceptable given the significantly higher failure rate of condoms compared with hormonal contraceptives,^35^ such trials may be limited to head-to-head studies of hormonal contraceptives to yield those formulations with the smallest adverse effect on the affective state, if any. The availability of such data would not only improve the generalisability of the findings to this first-time user group, but also provide more precise estimates. This is essential as the reported effect size in this study was still of very low certainty of evidence, according to the GRADE criteria for network meta-analysis, mainly because of the limited number of trials per comparison and the limited sample sizes in the trials.

This study has some major strengths. The project is the first network meta-analysis on the effect of hormonal contraceptives on depressive symptoms, and extends previous work that qualitatively reviewed observational and experimental studies.^5,6,33^ Moreover, it is substantially more comprehensive than previous reviews, as it includes seven trials that were not incorporated previously^19,21,23–27^ and includes data from three trials that have not been published before.^19,20,26^ Nevertheless, this study has also some limitations. Only one or a few trials were available for each comparison, and many trials had a relatively small sample size, which is probably insufficient to detect small but clinically important effects on depressive symptoms for specific formulations. This limitation is reflected in our ratings of the certainty of evidence for each comparison. Also, the quality of our analysis is limited by inherent limitations of individual included trials. First, very few trials provided sufficient data on important moderators, making it difficult to assess whether the transitivity assumption for network meta-analysis held. Only a few studies included women that had previous negative experiences with hormonal contraceptive use, which may have created a sample that has been less vulnerable to depressive symptoms than the average population of women who use hormonal contraceptives. Second, only one trial used an observer-rated scale to assess depressive symptoms, whereas the other trials used self-reported questionnaires or a related concept (negative affect). Although self-reported rating scales are efficient in terms of time and costs, and provide valuable insights into the subjective experience of severity of symptoms, only observer-rated structured diagnostic assessment procedures can assess valid diagnoses of major depression disorders.

With our network meta-analysis, we provide quantitative evidence, based on experimental data, that hormonal contraceptive use is relatively safe regarding the effect on depressive symptoms, especially in adult women. However, few trials were available for each formulation of hormonal contraceptives, and certainty of evidence for each comparison was rated as very low. Only a limited number of first-time users or women with previously negative experiences with hormonal contraceptive use were included, which limits the generalisability of our findings to these groups. This implies that awareness of the possible existence of mood problems among women who use hormonal contraceptives remains critical to their health and well-being. Depression severely affects the functioning of the individual and the people around them,^35^ and it also reduces hormonal contraceptive use adherence.^36^ However, by no means do we suggest to limit hormonal contraceptive use to counterbalance this potential, but not proven, risk for depressive symptoms. Access to safe and effective methods of birth control is a basic human right, and many women benefit from hormonal contraceptive use, as it improves dysmenorrhea and reproductive autonomy.^1^ Moreover, hormonal contraceptive use is much safer than pregnancy and associated postpartum depression risks.^37^ More RCTs investigating the safety of hormonal contraceptive use, that include depressive symptoms as one of their outcomes, are warranted to help women and their doctors make better-informed choices when deciding among possible methods of contraception.

## Data Availability

Upon publication of this article, the full data-set and script for analysing the data will be freely available from https://osf.io/2ehyw/.

## Supplementary material

For supplementary material accompanying this paper visit http://dx.doi.org/10.1192/bjo.2021.64. click here to view supplementary material
