# Supplementary material for: Hormonal contraceptive use and depressive symptoms: systematic review and network meta-analysis of randomised trials
Source: BJPsych Open. 2021 Jun 8;7(4):e110. doi: 10.1192/bjo.2021.64 (PMC8220855; doi:10.1192/bjo.2021.64)
Supplement: Supplementary file 1 [file S2056472421000648sup001.docx]

**Data supplement to:**

**Hormonal contraceptive use and depressive symptoms: systematic review and network meta-analysis of randomized trials**

Contents

[Contents 1](#_Toc67477162)

[Supplement 1. Search strings 2](#_Toc67477163)

[Supplement 2. Quality assessment: GRADE for the effects of hormonal contraceptives on depressive symptoms 10](#_Toc67477164)

[Supplement 2 Table 1. Overall judgement of the certainty of evidence per network estimate. 10](#_Toc67477165)

[1. Within study bias 11](#_Toc67477166)

[Supplement 2 Table 2. Study specific risk of bias judgements 12](#_Toc67477167)

[Supplement 2 Figure 1. Network estimate specific risk of bias judgements 13](#_Toc67477168)

[2. Reporting bias 14](#_Toc67477169)

[Supplement 2 Figure 2. Funnel plot of randomized clinical trials of hormonal contraceptives versus placebo 14](#_Toc67477170)

[3. Indirectness 14](#_Toc67477171)

[Supplement 2 Figure 3. Network estimate specific indirectness 16](#_Toc67477172)

[4. Imprecision 17](#_Toc67477173)

[5. Heterogeneity 17](#_Toc67477174)

[6. Incoherence 17](#_Toc67477175)

[Supplement 2 Figure 4. Estimates of direct and indirect effects 18](#_Toc67477176)

[References 19](#_Toc67477177)

### Supplement 1. Search strings

The search includes searching terms for the intervention (hormonal contraceptive), the outcome (depression or depressive symptoms), the design (randomized clinical trial), and the participants (female sex).

**Search conducted in Pubmed.**

(("synthetic estrogen"[tw] OR "synthetic estrogens"[tw] OR "synthetic oestrogen"[tw] OR "synthetic oestrogens"[tw] OR "Estradiol Congeners"[Mesh] OR "2- Methoxyestradiol"[tw] OR "Alkylated Estrogenic Steroids"[tw] OR "Catechol Estrogens"[tw] OR "Conjugated Estrogens"[tw] OR "Equilenin"[tw] OR "Equilin"[tw] OR "Esterified Estrogens"[tw] OR "Estetrol"[tw] OR "Estradiol"[tw] OR "Estriol"[tw] OR "Estrone"[tw] OR "Ethinyl Estradiol"[tw] OR "Ethinyl Estradiol-Norgestrel Combination"[tw] OR "Fulvestrant"[tw] OR "Hydroxyestrones"[tw] OR "Mestranol"[tw] OR "Quinestrol"[tw] OR "Catechol Estrogen"[tw] OR "Conjugated Estrogen"[tw] OR "Esterified Estrogen"[tw] OR "Hydroxyestrone"[tw] OR "Catechol oestrogen"[tw] OR "Conjugated oestrogen"[tw] OR "Esterified oestrogen"[tw] OR "Catechol oestrogen"[tw] OR "Conjugated oestrogen"[tw] OR "Esterified oestrogen"[tw] OR "Estrogen Replacement Therapy"[Mesh] OR "Estrogen Replacement"[tw] OR "Oestrogen Replacement"[tw] OR "Hormone Replacement"[tw] OR "Progestin Replacement"[tw] OR "Contraceptives, Oral, Hormonal"[Mesh] OR "Contraceptives, Oral, Hormonal"[Pharmacological Action] OR "Contraceptive Agents, Hormonal"[Mesh] OR "Contraceptive Agents, Hormonal"[Pharmacological Action] OR "Contraceptive Agents, Female" [mesh] OR "Contraceptive Agents, Female" [Pharmacological Action] OR "Algestone Acetophenide"[mesh] OR "Algestone Acetophenide"[tw] OR "Algestone"[mesh] OR "Algestone"[tw] OR "anordiol"[Supplementary Concept] OR "anordiol"[tw] OR "anordrin"[Supplementary Concept] OR "anordrin"[tw] OR "Bisecurin I"[Supplementary Concept] OR "Bisecurin I"[tw] OR "Centchroman"[mesh] OR "Centchroman"[tw] OR "Chlormadinone Acetate"[mesh] OR "Chlormadinone Acetate"[tw] OR "Cloprostenol"[mesh] OR "Cloprostenol"[tw] OR "CycloProvera"[Supplementary Concept] OR "CycloProvera"[tw] OR "Cyklabil"[Supplementary Concept] OR "Cyklabil"[tw] OR "Deposiston"[Supplementary Concept] OR "Deposiston"[tw] OR "Desogestrel"[mesh] OR "Desogestrel"[tw] OR "diallyl disulfide"[supplementary concept] OR "diallyl disulfide"[tw] OR "diallyl trisulfide"[supplementary concept] OR "diallyl trisulfide"[tw] OR "dienogest"[Supplementary Concept] OR "dienogest"[tw] OR "Dimethisterone"[mesh] OR "Dimethisterone"[tw] OR "estradiol 17 beta-cypionate"[Supplementary Concept] OR "estradiol 17 beta-cypionate"[tw] OR "estradiol 3-benzoate"[Supplementary Concept] OR "estradiol 3-benzoate"[tw] OR "estropipate"[Supplementary Concept] OR "estropipate"[tw] OR "Ethinyl Estradiol"[mesh] OR "Ethinyl Estradiol"[tw] OR "ethinyl estradiol, levonorgestrel drug combination"[Supplementary Concept] OR "ethinyl estradiol, levonorgestrel drug combination"[tw] OR "Ethinyl Estradiol-Norgestrel Combination"[mesh] OR "Ethinyl Estradiol-Norgestrel Combination"[tw] OR "Ethynodiol Diacetate"[mesh] OR "Ethynodiol Diacetate"[tw] OR "etonogestrel"[Supplementary Concept] OR "etonogestrel"[tw] OR "Femovan"[supplementary concept] OR "Femovan"[tw] OR "fluprostenol"[Supplementary Concept] OR "fluprostenol"[tw] OR "Gestodene"[Supplementary Concept] OR "Gestodene"[tw] OR "Gestrinone"[mesh] OR "Gestrinone"[tw] OR "gossypol acetic acid"[supplementary concept] OR "gossypol acetic acid"[tw] OR "Infecundin"[Supplementary Concept] OR "Infecundin"[tw] OR "levonorgestrel butanoate"[Supplementary Concept] OR "levonorgestrel butanoate"[tw] OR "Levonorgestrel"[mesh] OR "Levonorgestrel"[tw] OR "Lynestrenol"[mesh] OR "Lynestrenol"[tw] OR "Medroxyprogesterone Acetate"[mesh] OR "Medroxyprogesterone Acetate"[tw] OR "Medroxyprogesterone"[mesh] OR "Medroxyprogesterone"[tw] OR "Megestrol"[mesh] OR "Megestrol"[tw] OR "Mestranol"[mesh] OR "Mestranol"[tw] OR "Mifepristone"[mesh] OR "Mifepristone"[tw] OR "neem oil"[supplementary concept] OR "neem oil"[tw] OR "Nonidet P-40"[supplementary concept] OR "Nonidet P-40"[tw] OR "Non- Ovlon"[Supplementary Concept] OR "Non-Ovlon"[tw] OR "Nonoxynol"[mesh] OR "Nonoxynol"[tw] OR "Noracycline"[Supplementary Concept] OR "Noracycline"[tw] OR "norelgestromin"[Supplementary Concept] OR "norelgestromin"[tw] OR "Norethindrone Acetate"[mesh] OR "Norethindrone Acetate"[tw] OR "norethindrone acetate, ethinyl estradiol, ferrous fumarate drug combination"[Supplementary Concept] OR "norethindrone acetate, ethinyl estradiol, ferrous fumarate drug combination"[tw] OR "norethindrone enanthate"[Supplementary Concept] OR "norethindrone enanthate"[tw] OR "Norethindrone"[mesh] OR "Norethindrone"[tw] OR "Norethynodrel"[mesh] OR "Norethynodrel"[tw] OR "norgestimate"[Supplementary Concept] OR "norgestimate"[tw] OR "norgestimate, ethinyl estradiol drug combination"[Supplementary Concept] OR "norgestimate, ethinyl estradiol drug combination"[tw] OR "Norgestrel"[mesh] OR "Norgestrel"[tw] OR "Norgestrienone"[mesh] OR "Norgestrienone"[tw] OR "Norinyl"[Supplementary Concept] OR "Norinyl"[tw] OR "NuvaRing"[Supplementary Concept] OR "NuvaRing"[tw] OR "Octoxynol"[mesh] OR "Octoxynol"[tw] OR "Ortho Evra"[Supplementary Concept] OR "Ortho Evra"[tw] OR "Oviol"[Supplementary Concept] OR "Oviol"[tw] OR "SC-11800 EE"[Supplementary Concept] OR "SC-11800 EE"[tw] OR "ST 1435"[Supplementary Concept] OR "ST 1435"[tw] OR "sulprostone"[Supplementary Concept] OR "sulprostone"[tw] OR "trestolone"[Supplementary Concept] OR "trestolone"[tw] OR "Triptorelin Pamoate"[mesh] OR "Triptorelin Pamoate"[tw] OR "Trisequens"[Supplementary Concept] OR "Trisequens"[tw] OR "ulipristal acetate"[Supplementary Concept] OR "ulipristal acetate"[tw] OR "Estrogens"[Mesh] OR "Estrogens"[Pharmacological Action] OR "estrogen"[tw] OR "estrogens"[tw] OR estrogen*[tw] OR "oestrogen"[tw] OR "oestrogens"[tw] OR oestrogen*[tw] OR "2,3-bis(3'-hydroxybenzyl)butane-1,4- diol"[Supplementary Concept] OR "2,3-bis(3'- hydroxybenzyl)butyrolactone"[Supplementary Concept] OR "4- octylphenol"[Supplementary Concept] OR "8-prenylnaringenin"[Supplementary Concept] OR "biochanin A"[Supplementary Concept] OR "bisphenol A"[Supplementary Concept] OR "Chlorotrianisene"[mesh] OR "Coumestrol"[mesh] OR "daidzein"[Supplementary Concept] OR "Dienestrol"[mesh] OR "Diethylstilbestrol"[mesh] OR "diethylstilbestrol dipropionate"[Supplementary Concept] OR "Epimestrol"[mesh] OR "Equol"[mesh] OR "Estradiol"[mesh] OR "estradiol enanthate"[Supplementary Concept] OR "Estrogenic Steroids, Alkylated"[mesh] OR "Estrogens, Conjugated (USP)"[mesh] OR "estrogens, conjugated synthetic A"[Supplementary Concept] OR "estrogens, conjugated synthetic B"[Supplementary Concept] OR "Estrogens, Esterified (USP)"[mesh] OR "Estrone"[mesh] OR "Ethinyl Estradiol"[mesh] OR "formononetin"[Supplementary Concept] OR "Genistein"[mesh] OR "glycitein"[Supplementary Concept] OR "glycitin"[Supplementary Concept] OR "Hexestrol"[mesh] OR "Infecundin"[Supplementary Concept] OR "Mestranol"[mesh] OR "nylestriol"[Supplementary Concept] OR "o,p'-DDT"[Supplementary Concept] OR "O- desmethylangolensin"[Supplementary Concept] OR "polyestradiol phosphate"[Supplementary Concept] OR "Quinestrol"[mesh] OR "secoisolariciresinol"[Supplementary Concept] OR "Zearalenone"[mesh] OR "Zeranol"[mesh] OR "2,3-bis(3'-hydroxybenzyl)butane-1,4-diol"[tw] OR "2,3-bis(3'- hydroxybenzyl)butyrolactone"[tw] OR "4-octylphenol"[tw] OR "8-prenylnaringenin"[tw] OR "biochanin A"[tw] OR "bisphenol A"[tw] OR "Chlorotrianisene"[tw] OR "Coumestrol"[tw] OR "daidzein"[tw] OR "Dienestrol"[tw] OR "Diethylstilbestrol"[tw] OR "diethylstilbestrol dipropionate"[tw] OR "Epimestrol"[tw] OR "Equol"[tw] OR "Estradiol"[tw] OR "estradiol enanthate"[tw] OR "Estrogenic Steroids, Alkylated"[tw] OR "Estrogens, Conjugated (USP)"[tw] OR "estrogens, conjugated synthetic A"[tw] OR "estrogens, conjugated synthetic B"[tw] OR "Estrogens, Esterified (USP)"[tw] OR "Estrone"[tw] OR "Ethinyl Estradiol"[tw] OR "formononetin"[tw] OR "Genistein"[tw] OR "glycitein"[tw] OR "glycitin"[tw] OR "Hexestrol"[tw] OR "Infecundin"[tw] OR "Mestranol"[tw] OR "nylestriol"[tw] OR "o,p'-DDT"[tw] OR "O- desmethylangolensin"[tw] OR "polyestradiol phosphate"[tw] OR "Quinestrol"[tw] OR "secoisolariciresinol"[tw] OR "Zearalenone"[tw] OR "Zeranol"[tw]) **AND** ("Depression"[Mesh] OR "Depressive Disorder"[Mesh] OR "depression"[tw] OR "depressive"[tw] OR "Mood Disorders"[Mesh] OR "Affect"[Mesh] OR "mood"[tw] OR "Cyclothymic Disorder"[tw] OR "Dysthymic Disorder"[tw] OR "Premenstrual Dysphoric Disorder"[tw] OR "Seasonal Affective Disorder"[tw] OR "Cyclothymic Disorders"[tw] OR "Dysthymic Disorders"[tw] OR "Premenstrual Dysphoric Disorders"[tw] OR "Seasonal Affective Disorders"[tw] OR "Premenstrual Syndrome"[mesh] OR "premenstrual syndrome"[tw] OR premenstrual symptom*[tw] OR menstrual symptom*[tw] OR "premenstrual mood"[tw] OR "intermenstrual mood"[tw] OR "menstrual mood"[tw]) NOT (("Male"[mesh] OR "Men"[mesh] OR "male"[ti] OR "males"[ti] OR "men"[ti]) NOT ("Female"[mesh] OR "Women"[mesh] OR "Female"[ti] OR "Women"[ti] OR "girl"[ti] OR "girls"[ti] OR "girlhood"[ti])) NOT (("Infant"[mesh] OR "Child"[mesh]) NOT ("Adolescent"[mesh] OR "Adult"[mesh])) NOT ("Animals"[mesh] NOT "Humans"[mesh]) **AND** english[la] **AND** ("clinical trial"[pt] OR "clinical trial"[tiab] OR "clinical trials as topic"[mesh] OR "clinical trials"[tiab] OR "control groups"[mesh] OR "control group"[tiab] OR "control groups"[tiab] OR "controlled clinical trial"[pt] OR "controlled clinical trials as topic"[mesh] OR "cross- over studies"[mesh] OR "cross over study"[tiab] OR "cross over studies"[tiab] OR "double-blind method"[mesh] OR "double blind"[tiab] OR "evaluation studies as topic"[mesh] OR "follow-up studies"[mesh] OR "follow up study"[tiab] OR "follow up studies"[tiab] OR "placebos"[mesh] OR placebo*[tiab] OR placebos*[tiab] OR "pragmatic clinical trial"[pt] OR "prospective studies"[mesh] OR "prospective study"[tiab] OR "prospective studies"[tiab] OR "RaCT"[tiab] OR "RaCTs"[tiab] OR "random allocation"[mesh] OR "randomised "[tiab] OR "randomized controlled trial"[pt] OR "randomized controlled trials as topic"[mesh] OR "randomized"[tiab] OR random*[tiab] OR "RCT"[tiab] OR "RCTs"[tiab] OR "Research Design"[MeSH:noexp] OR "Research design"[tiab] OR "Research designs"[tiab] OR "single blind"[tiab] OR "single-blind method"[mesh] OR ((single*[tiab] OR double*[tiab] OR triple*[tiab]) **AND** (blind*[tiab] OR mask*[tiab])) OR volunteer*[tiab] OR "trial"[ti] OR "trials"[ti]))

**Search conducted in Embase**

((*"ethinylestradiol"/ OR exp *"estrogen"/ OR exp *"estrogen therapy"/ OR exp *"oral contraceptive agent"/ OR exp *"hormonal contraception"/ OR "synthetic estrogen".ti,ab OR "synthetic estrogens".ti,ab OR "synthetic oestrogen".ti,ab OR "synthetic oestrogens".ti,ab OR "2-Methoxyestradiol".ti,ab OR "Alkylated Estrogenic Steroids".ti,ab OR "Catechol Estrogens".ti,ab OR "Conjugated Estrogens".ti,ab OR "Equilenin".ti,ab OR "Equilin".ti,ab OR "Esterified Estrogens".ti,ab OR "Estetrol".ti,ab OR "Estradiol".ti,ab OR "Estriol".ti,ab OR "Estrone".ti,ab OR "Ethinyl Estradiol".ti,ab OR "Ethinyl Estradiol-Norgestrel Combination".ti,ab OR "Fulvestrant".ti,ab OR "Hydroxyestrones".ti,ab OR "Mestranol".ti,ab OR "Quinestrol".ti,ab OR "Catechol Estrogen".ti,ab OR "Conjugated Estrogen".ti,ab OR "Esterified Estrogen".ti,ab OR "Hydroxyestrone".ti,ab OR "Catechol oestrogen".ti,ab OR "Conjugated oestrogen".ti,ab OR "Esterified oestrogen".ti,ab OR "Catechol oestrogen".ti,ab OR "Conjugated oestrogen".ti,ab OR "Esterified oestrogen".ti,ab OR "Estrogen Replacement".ti,ab OR "Oestrogen Replacement".ti,ab OR "Hormone Replacement".ti,ab OR "Progestin Replacement".ti,ab OR "Algestone Acetophenide".ti,ab OR "Algestone Acetophenide".ti,ab OR "Algestone".ti,ab OR "Algestone".ti,ab OR "anordiol".ti,ab OR "anordiol".ti,ab OR "anordrin".ti,ab OR "anordrin".ti,ab OR "Bisecurin I".ti,ab OR "Bisecurin I".ti,ab OR "Centchroman".ti,ab OR "Centchroman".ti,ab OR "Chlormadinone Acetate".ti,ab OR "Chlormadinone Acetate".ti,ab OR "Cloprostenol".ti,ab OR "Cloprostenol".ti,ab OR "CycloProvera".ti,ab OR "CycloProvera".ti,ab OR "Cyklabil".ti,ab OR "Cyklabil".ti,ab OR "Deposiston".ti,ab OR "Deposiston".ti,ab OR "Desogestrel".ti,ab OR "Desogestrel".ti,ab OR "diallyl disulfide".ti,ab OR "diallyl disulfide".ti,ab OR "diallyl trisulfide".ti,ab OR "diallyl trisulfide".ti,ab OR "dienogest".ti,ab OR "dienogest".ti,ab OR "Dimethisterone".ti,ab OR "Dimethisterone".ti,ab OR "estradiol 17 beta-cypionate".ti,ab OR "estradiol 17 beta-cypionate".ti,ab OR "estradiol 3-benzoate".ti,ab OR "estradiol 3-benzoate".ti,ab OR "estropipate".ti,ab OR "estropipate".ti,ab OR "Ethinyl Estradiol".ti,ab OR "Ethinyl Estradiol".ti,ab OR "ethinyl estradiol, levonorgestrel drug combination".ti,ab OR "ethinyl estradiol, levonorgestrel drug combination".ti,ab OR "Ethinyl Estradiol-Norgestrel Combination".ti,ab OR "Ethinyl Estradiol-Norgestrel Combination".ti,ab OR "Ethynodiol Diacetate".ti,ab OR "Ethynodiol Diacetate".ti,ab OR "etonogestrel".ti,ab OR "etonogestrel".ti,ab OR "Femovan".ti,ab OR "Femovan".ti,ab OR "fluprostenol".ti,ab OR "fluprostenol".ti,ab OR "Gestodene".ti,ab OR "Gestodene".ti,ab OR "Gestrinone".ti,ab OR "Gestrinone".ti,ab OR "gossypol acetic acid".ti,ab OR "gossypol acetic acid".ti,ab OR "Infecundin".ti,ab OR "Infecundin".ti,ab OR "levonorgestrel butanoate".ti,ab OR "levonorgestrel butanoate".ti,ab OR "Levonorgestrel".ti,ab OR "Levonorgestrel".ti,ab OR "Lynestrenol".ti,ab OR "Lynestrenol".ti,ab OR "Medroxyprogesterone Acetate".ti,ab OR "Medroxyprogesterone Acetate".ti,ab OR "Medroxyprogesterone".ti,ab OR "Medroxyprogesterone".ti,ab OR "Megestrol".ti,ab OR "Megestrol".ti,ab OR "Mestranol".ti,ab OR "Mestranol".ti,ab OR "Mifepristone".ti,ab OR "Mifepristone".ti,ab OR "neem oil".ti,ab OR "neem oil".ti,ab OR "Nonidet P-40".ti,ab OR "Nonidet P-40".ti,ab OR "Non-Ovlon".ti,ab OR "Non-Ovlon".ti,ab OR "Nonoxynol".ti,ab OR "Nonoxynol".ti,ab OR "Noracycline".ti,ab OR "Noracycline".ti,ab OR "norelgestromin".ti,ab OR "norelgestromin".ti,ab OR "Norethindrone Acetate".ti,ab OR "Norethindrone Acetate".ti,ab OR "norethindrone acetate, ethinyl estradiol, ferrous fumarate drug combination".ti,ab OR "norethindrone acetate, ethinyl estradiol, ferrous fumarate drug combination".ti,ab OR "norethindrone enanthate".ti,ab OR "norethindrone enanthate".ti,ab OR "Norethindrone".ti,ab OR "Norethindrone".ti,ab OR "Norethynodrel".ti,ab OR "Norethynodrel".ti,ab OR "norgestimate".ti,ab OR "norgestimate".ti,ab OR "norgestimate, ethinyl estradiol drug combination".ti,ab OR "norgestimate, ethinyl estradiol drug combination".ti,ab OR "Norgestrel".ti,ab OR "Norgestrel".ti,ab OR "Norgestrienone".ti,ab OR "Norgestrienone".ti,ab OR "Norinyl".ti,ab OR "Norinyl".ti,ab OR "NuvaRing".ti,ab OR "NuvaRing".ti,ab OR "Octoxynol".ti,ab OR "Octoxynol".ti,ab OR "Ortho Evra".ti,ab OR "Ortho Evra".ti,ab OR "Oviol".ti,ab OR "Oviol".ti,ab OR "SC-11800 EE".ti,ab OR "SC-11800 EE".ti,ab OR "ST 1435".ti,ab OR "ST 1435".ti,ab OR "sulprostone".ti,ab OR "sulprostone".ti,ab OR "trestolone".ti,ab OR "trestolone".ti,ab OR "Triptorelin Pamoate".ti,ab OR "Triptorelin Pamoate".ti,ab OR "Trisequens".ti,ab OR "Trisequens".ti,ab OR "ulipristal acetate".ti,ab OR "ulipristal acetate".ti,ab OR "estrogen".ti,ab OR "estrogens".ti,ab OR estrogen*.ti,ab OR "oestrogen".ti,ab OR "oestrogens".ti,ab OR oestrogen*.ti,ab OR "2,3-bis(3'-hydroxybenzyl)butane-1,4-diol".ti,ab OR "2,3-bis(3'-hydroxybenzyl)butyrolactone".ti,ab OR "4-octylphenol".ti,ab OR "8-prenylnaringenin".ti,ab OR "biochanin A".ti,ab OR "bisphenol A".ti,ab OR "Chlorotrianisene".ti,ab OR "Coumestrol".ti,ab OR "daidzein".ti,ab OR "Dienestrol".ti,ab OR "Diethylstilbestrol".ti,ab OR "diethylstilbestrol dipropionate".ti,ab OR "Epimestrol".ti,ab OR "Equol".ti,ab OR "Estradiol".ti,ab OR "estradiol enanthate".ti,ab OR "Estrogenic Steroids, Alkylated".ti,ab OR "Estrogens, Conjugated (USP)".ti,ab OR "estrogens, conjugated synthetic A".ti,ab OR "estrogens, conjugated synthetic B".ti,ab OR "Estrogens, Esterified (USP)".ti,ab OR "Estrone".ti,ab OR "Ethinyl Estradiol".ti,ab OR "formononetin".ti,ab OR "Genistein".ti,ab OR "glycitein".ti,ab OR "glycitin".ti,ab OR "Hexestrol".ti,ab OR "Infecundin".ti,ab OR "Mestranol".ti,ab OR "nylestriol".ti,ab OR "o,p'-DDT".ti,ab OR "O-desmethylangolensin".ti,ab OR "polyestradiol phosphate".ti,ab OR "Quinestrol".ti,ab OR "secoisolariciresinol".ti,ab OR "Zearalenone".ti,ab OR "Zeranol".ti,ab OR "2,3-bis(3'-hydroxybenzyl)butane-1,4-diol".ti,ab OR "2,3-bis(3'-hydroxybenzyl)butyrolactone".ti,ab OR "4-octylphenol".ti,ab OR "8-prenylnaringenin".ti,ab OR "biochanin A".ti,ab OR "bisphenol A".ti,ab OR "Chlorotrianisene".ti,ab OR "Coumestrol".ti,ab OR "daidzein".ti,ab OR "Dienestrol".ti,ab OR "Diethylstilbestrol".ti,ab OR "diethylstilbestrol dipropionate".ti,ab OR "Epimestrol".ti,ab OR "Equol".ti,ab OR "Estradiol".ti,ab OR "estradiol enanthate".ti,ab OR "Estrogenic Steroids, Alkylated".ti,ab OR "Estrogens, Conjugated (USP)".ti,ab OR "estrogens, conjugated synthetic A".ti,ab OR "estrogens, conjugated synthetic B".ti,ab OR "Estrogens, Esterified (USP)".ti,ab OR "Estrone".ti,ab OR "Ethinyl Estradiol".ti,ab OR "formononetin".ti,ab OR "Genistein".ti,ab OR "glycitein".ti,ab OR "glycitin".ti,ab OR "Hexestrol".ti,ab OR "Infecundin".ti,ab OR "Mestranol".ti,ab OR "nylestriol".ti,ab OR "o,p'-DDT".ti,ab OR "O-desmethylangolensin".ti,ab OR "polyestradiol phosphate".ti,ab OR "Quinestrol".ti,ab OR "secoisolariciresinol".ti,ab OR "Zearalenone".ti,ab OR "Zeranol".ti,ab) AND (exp *"Depression"/ OR "depression".ti,ab OR "depressive".ti,ab OR exp *"Mood Disorder"/ OR exp *"Mood"/ OR "mood".ti,ab OR "Cyclothymic Disorder".ti,ab OR "Dysthymic Disorder".ti,ab OR "Premenstrual Dysphoric Disorder".ti,ab OR "Seasonal Affective Disorder".ti,ab OR "Cyclothymic Disorders".ti,ab OR "Dysthymic Disorders".ti,ab OR "Premenstrual Dysphoric Disorders".ti,ab OR "Seasonal Affective Disorders".ti,ab OR *"Premenstrual Syndrome"/ OR "premenstrual syndrome".ti,ab OR "premenstrual symptom*".ti,ab OR "menstrual symptom*".ti,ab OR "premenstrual mood".ti,ab OR "intermenstrual mood".ti,ab OR "menstrual mood".ti,ab) NOT ((exp "Male"/ OR exp "Men"/ OR "male".ti OR "males".ti OR "men".ti) NOT (exp "Female"/ OR exp "Women"/ OR "Female".ti OR "Women".ti OR "girl".ti OR "girls".ti OR "girlhood".ti)) NOT ((exp "Infant"/ OR exp "Child"/) NOT (exp "Adolescent"/ OR exp "Adult"/)) NOT (exp "Animals"/ NOT exp "Humans"/) AND english.la AND (exp "clinical trial"/ OR "clinical trial".ti,ab OR "clinical trials".ti,ab OR "placebo"/ OR placebo*.ti,ab OR placebos*.ti,ab OR "RaCT".ti,ab OR "RaCTs".ti,ab OR "randomization"/ OR random*.ti,ab OR "RCT".ti,ab OR "RCTs".ti,ab OR ((single*.ti,ab OR double*.ti,ab OR triple*.ti,ab) ADJ5 (blind*.ti,ab OR mask*.ti,ab)) OR volunteer*.ti,ab OR "trial".ti OR "trials".ti)) NOT (conference review or conference abstract).pt

**Search conducted in Web of Science**

(ts=("ethinylestradiol" OR "estrogen" OR "estrogen therapy" OR "oral contraceptive agent" OR "hormonal contraception" OR "synthetic estrogen" OR "synthetic estrogens" OR "synthetic oestrogen" OR "synthetic oestrogens" OR "2-Methoxyestradiol" OR "Alkylated Estrogenic Steroids" OR "Catechol Estrogens" OR "Conjugated Estrogens" OR "Equilenin" OR "Equilin" OR "Esterified Estrogens" OR "Estetrol" OR "Estradiol" OR "Estriol" OR "Estrone" OR "Ethinyl Estradiol" OR "Ethinyl Estradiol-Norgestrel Combination" OR "Fulvestrant" OR "Hydroxyestrones" OR "Mestranol" OR "Quinestrol" OR "Catechol Estrogen" OR "Conjugated Estrogen" OR "Esterified Estrogen" OR "Hydroxyestrone" OR "Catechol oestrogen" OR "Conjugated oestrogen" OR "Esterified oestrogen" OR "Catechol oestrogen" OR "Conjugated oestrogen" OR "Esterified oestrogen" OR "Estrogen Replacement" OR "Oestrogen Replacement" OR "Hormone Replacement" OR "Progestin Replacement" OR "Algestone Acetophenide" OR "Algestone Acetophenide" OR "Algestone" OR "Algestone" OR "anordiol" OR "anordiol" OR "anordrin" OR "anordrin" OR "Bisecurin I" OR "Bisecurin I" OR "Centchroman" OR "Centchroman" OR "Chlormadinone Acetate" OR "Chlormadinone Acetate" OR "Cloprostenol" OR "Cloprostenol" OR "CycloProvera" OR "CycloProvera" OR "Cyklabil" OR "Cyklabil" OR "Deposiston" OR "Deposiston" OR "Desogestrel" OR "Desogestrel" OR "diallyl disulfide" OR "diallyl disulfide" OR "diallyl trisulfide" OR "diallyl trisulfide" OR "dienogest" OR "dienogest" OR "Dimethisterone" OR "Dimethisterone" OR "estradiol 17 beta-cypionate" OR "estradiol 17 beta-cypionate" OR "estradiol 3-benzoate" OR "estradiol 3-benzoate" OR "estropipate" OR "estropipate" OR "Ethinyl Estradiol" OR "Ethinyl Estradiol" OR "ethinyl estradiol, levonorgestrel drug combination" OR "ethinyl estradiol, levonorgestrel drug combination" OR "Ethinyl Estradiol-Norgestrel Combination" OR "Ethinyl Estradiol-Norgestrel Combination" OR "Ethynodiol Diacetate" OR "Ethynodiol Diacetate" OR "etonogestrel" OR "etonogestrel" OR "Femovan" OR "Femovan" OR "fluprostenol" OR "fluprostenol" OR "Gestodene" OR "Gestodene" OR "Gestrinone" OR "Gestrinone" OR "gossypol acetic acid" OR "gossypol acetic acid" OR "Infecundin" OR "Infecundin" OR "levonorgestrel butanoate" OR "levonorgestrel butanoate" OR "Levonorgestrel" OR "Levonorgestrel" OR "Lynestrenol" OR "Lynestrenol" OR "Medroxyprogesterone Acetate" OR "Medroxyprogesterone Acetate" OR "Medroxyprogesterone" OR "Medroxyprogesterone" OR "Megestrol" OR "Megestrol" OR "Mestranol" OR "Mestranol" OR "Mifepristone" OR "Mifepristone" OR "neem oil" OR "neem oil" OR "Nonidet P-40" OR "Nonidet P-40" OR "Non-Ovlon" OR "Non-Ovlon" OR "Nonoxynol" OR "Nonoxynol" OR "Noracycline" OR "Noracycline" OR "norelgestromin" OR "norelgestromin" OR "Norethindrone Acetate" OR "Norethindrone Acetate" OR "norethindrone acetate, ethinyl estradiol, ferrous fumarate drug combination" OR "norethindrone acetate, ethinyl estradiol, ferrous fumarate drug combination" OR "norethindrone enanthate" OR "norethindrone enanthate" OR "Norethindrone" OR "Norethindrone" OR "Norethynodrel" OR "Norethynodrel" OR "norgestimate" OR "norgestimate" OR "norgestimate, ethinyl estradiol drug combination" OR "norgestimate, ethinyl estradiol drug combination" OR "Norgestrel" OR "Norgestrel" OR "Norgestrienone" OR "Norgestrienone" OR "Norinyl" OR "Norinyl" OR "NuvaRing" OR "NuvaRing" OR "Octoxynol" OR "Octoxynol" OR "Ortho Evra" OR "Ortho Evra" OR "Oviol" OR "Oviol" OR "SC-11800 EE" OR "SC-11800 EE" OR "ST 1435" OR "ST 1435" OR "sulprostone" OR "sulprostone" OR "trestolone" OR "trestolone" OR "Triptorelin Pamoate" OR "Triptorelin Pamoate" OR "Trisequens" OR "Trisequens" OR "ulipristal acetate" OR "ulipristal acetate" OR "estrogen" OR "estrogens" OR estrogen* OR "oestrogen" OR "oestrogens" OR oestrogen* OR "2,3-bis(3'-hydroxybenzyl)butane-1,4-diol" OR "2,3-bis(3'-hydroxybenzyl)butyrolactone" OR "4-octylphenol" OR "8-prenylnaringenin" OR "biochanin A" OR "bisphenol A" OR "Chlorotrianisene" OR "Coumestrol" OR "daidzein" OR "Dienestrol" OR "Diethylstilbestrol" OR "diethylstilbestrol dipropionate" OR "Epimestrol" OR "Equol" OR "Estradiol" OR "estradiol enanthate" OR "Estrogenic Steroids, Alkylated" OR "Estrogens, Conjugated (USP)" OR "estrogens, conjugated synthetic A" OR "estrogens, conjugated synthetic B" OR "Estrogens, Esterified (USP)" OR "Estrone" OR "Ethinyl Estradiol" OR "formononetin" OR "Genistein" OR "glycitein" OR "glycitin" OR "Hexestrol" OR "Infecundin" OR "Mestranol" OR "nylestriol" OR "o,p'-DDT" OR "O-desmethylangolensin" OR "polyestradiol phosphate" OR "Quinestrol" OR "secoisolariciresinol" OR "Zearalenone" OR "Zeranol" OR "2,3-bis(3'-hydroxybenzyl)butane-1,4-diol" OR "2,3-bis(3'-hydroxybenzyl)butyrolactone" OR "4-octylphenol" OR "8-prenylnaringenin" OR "biochanin A" OR "bisphenol A" OR "Chlorotrianisene" OR "Coumestrol" OR "daidzein" OR "Dienestrol" OR "Diethylstilbestrol" OR "diethylstilbestrol dipropionate" OR "Epimestrol" OR "Equol" OR "Estradiol" OR "estradiol enanthate" OR "Estrogenic Steroids, Alkylated" OR "Estrogens, Conjugated (USP)" OR "estrogens, conjugated synthetic A" OR "estrogens, conjugated synthetic B" OR "Estrogens, Esterified (USP)" OR "Estrone" OR "Ethinyl Estradiol" OR "formononetin" OR "Genistein" OR "glycitein" OR "glycitin" OR "Hexestrol" OR "Infecundin" OR "Mestranol" OR "nylestriol" OR "o,p'-DDT" OR "O-desmethylangolensin" OR "polyestradiol phosphate" OR "Quinestrol" OR "secoisolariciresinol" OR "Zearalenone" OR "Zeranol") AND ts=("Depression" OR "depression" OR "depressive" OR "Mood Disorder" OR "Mood" OR "mood" OR "Cyclothymic Disorder" OR "Dysthymic Disorder" OR "Premenstrual Dysphoric Disorder" OR "Seasonal Affective Disorder" OR "Cyclothymic Disorders" OR "Dysthymic Disorders" OR "Premenstrual Dysphoric Disorders" OR "Seasonal Affective Disorders" OR "Premenstrual Syndrome" OR "premenstrual syndrome" OR "premenstrual symptom*" OR "menstrual symptom*" OR "premenstrual mood" OR "intermenstrual mood" OR "menstrual mood") NOT ti=(("Male" OR "Men" OR "male" OR "males" OR "men") NOT ("Female" OR "Women" OR "Female" OR "Women" OR "girl" OR "girls" OR "girlhood")) NOT ti=(("Infant" OR "Child") NOT ("Adolescent" OR "Adult" OR "elderly" OR geriatr*)) NOT ti=("veterinary" OR "rabbit" OR "rabbits" OR "animal" OR "animals" OR "mouse" OR "mice" OR "rodent" OR "rodents" OR "rat" OR "rats" OR "pig" OR "pigs" OR "porcine" OR "horse" OR "horses" OR "equine" OR "cow" OR "cows" OR "bovine" OR "goat" OR "goats" OR "sheep" OR "ovine" OR "canine" OR "dog" OR "dogs" OR "feline" OR "cat" OR "cats") AND la=english AND (TS=("clinical trial" OR "clinical trial" OR "clinical trials" OR "placebo"/ OR placebo* OR placebos* OR "RaCT" OR "RaCTs" OR "randomization" OR random* OR "RCT" OR "RCTs" OR ((single* OR double* OR triple*) NEAR/5 (blind* OR mask*)) OR volunteer*) OR ti=("trial" OR "trials"))) NOT dt=(meeting abstract)

**Search conducted in Cochrane**

(("ethinylestradiol" OR "estrogen" OR "estrogen therapy" OR "oral contraceptive agent" OR "hormonal contraception" OR "synthetic estrogen" OR "synthetic estrogens" OR "synthetic oestrogen" OR "synthetic oestrogens" OR "2-Methoxyestradiol" OR "Alkylated Estrogenic Steroids" OR "Catechol Estrogens" OR "Conjugated Estrogens" OR "Equilenin" OR "Equilin" OR "Esterified Estrogens" OR "Estetrol" OR "Estradiol" OR "Estriol" OR "Estrone" OR "Ethinyl Estradiol" OR "Ethinyl Estradiol-Norgestrel Combination" OR "Fulvestrant" OR "Hydroxyestrones" OR "Mestranol" OR "Quinestrol" OR "Catechol Estrogen" OR "Conjugated Estrogen" OR "Esterified Estrogen" OR "Hydroxyestrone" OR "Catechol oestrogen" OR "Conjugated oestrogen" OR "Esterified oestrogen" OR "Catechol oestrogen" OR "Conjugated oestrogen" OR "Esterified oestrogen" OR "Estrogen Replacement" OR "Oestrogen Replacement" OR "Hormone Replacement" OR "Progestin Replacement" OR "Algestone Acetophenide" OR "Algestone Acetophenide" OR "Algestone" OR "Algestone" OR "anordiol" OR "anordiol" OR "anordrin" OR "anordrin" OR "Bisecurin I" OR "Bisecurin I" OR "Centchroman" OR "Centchroman" OR "Chlormadinone Acetate" OR "Chlormadinone Acetate" OR "Cloprostenol" OR "Cloprostenol" OR "CycloProvera" OR "CycloProvera" OR "Cyklabil" OR "Cyklabil" OR "Deposiston" OR "Deposiston" OR "Desogestrel" OR "Desogestrel" OR "diallyl disulfide" OR "diallyl disulfide" OR "diallyl trisulfide" OR "diallyl trisulfide" OR "dienogest" OR "dienogest" OR "Dimethisterone" OR "Dimethisterone" OR "estradiol 17 beta-cypionate" OR "estradiol 17 beta-cypionate" OR "estradiol 3-benzoate" OR "estradiol 3-benzoate" OR "estropipate" OR "estropipate" OR "Ethinyl Estradiol" OR "Ethinyl Estradiol" OR "ethinyl estradiol, levonorgestrel drug combination" OR "ethinyl estradiol, levonorgestrel drug combination" OR "Ethinyl Estradiol-Norgestrel Combination" OR "Ethinyl Estradiol-Norgestrel Combination" OR "Ethynodiol Diacetate" OR "Ethynodiol Diacetate" OR "etonogestrel" OR "etonogestrel" OR "Femovan" OR "Femovan" OR "fluprostenol" OR "fluprostenol" OR "Gestodene" OR "Gestodene" OR "Gestrinone" OR "Gestrinone" OR "gossypol acetic acid" OR "gossypol acetic acid" OR "Infecundin" OR "Infecundin" OR "levonorgestrel butanoate" OR "levonorgestrel butanoate" OR "Levonorgestrel" OR "Levonorgestrel" OR "Lynestrenol" OR "Lynestrenol" OR "Medroxyprogesterone Acetate" OR "Medroxyprogesterone Acetate" OR "Medroxyprogesterone" OR "Medroxyprogesterone" OR "Megestrol" OR "Megestrol" OR "Mestranol" OR "Mestranol" OR "Mifepristone" OR "Mifepristone" OR "neem oil" OR "neem oil" OR "Nonidet P-40" OR "Nonidet P-40" OR "Non-Ovlon" OR "Non-Ovlon" OR "Nonoxynol" OR "Nonoxynol" OR "Noracycline" OR "Noracycline" OR "norelgestromin" OR "norelgestromin" OR "Norethindrone Acetate" OR "Norethindrone Acetate" OR "norethindrone acetate, ethinyl estradiol, ferrous fumarate drug combination" OR "norethindrone acetate, ethinyl estradiol, ferrous fumarate drug combination" OR "norethindrone enanthate" OR "norethindrone enanthate" OR "Norethindrone" OR "Norethindrone" OR "Norethynodrel" OR "Norethynodrel" OR "norgestimate" OR "norgestimate" OR "norgestimate, ethinyl estradiol drug combination" OR "norgestimate, ethinyl estradiol drug combination" OR "Norgestrel" OR "Norgestrel" OR "Norgestrienone" OR "Norgestrienone" OR "Norinyl" OR "Norinyl" OR "NuvaRing" OR "NuvaRing" OR "Octoxynol" OR "Octoxynol" OR "Ortho Evra" OR "Ortho Evra" OR "Oviol" OR "Oviol" OR "SC-11800 EE" OR "SC-11800 EE" OR "ST 1435" OR "ST 1435" OR "sulprostone" OR "sulprostone" OR "trestolone" OR "trestolone" OR "Triptorelin Pamoate" OR "Triptorelin Pamoate" OR "Trisequens" OR "Trisequens" OR "ulipristal acetate" OR "ulipristal acetate" OR "estrogen" OR "estrogens" OR estrogen* OR "oestrogen" OR "oestrogens" OR oestrogen* OR "2,3-bis(3'-hydroxybenzyl)butane-1,4-diol" OR "2,3-bis(3'-hydroxybenzyl)butyrolactone" OR "4-octylphenol" OR "8-prenylnaringenin" OR "biochanin A" OR "bisphenol A" OR "Chlorotrianisene" OR "Coumestrol" OR "daidzein" OR "Dienestrol" OR "Diethylstilbestrol" OR "diethylstilbestrol dipropionate" OR "Epimestrol" OR "Equol" OR "Estradiol" OR "estradiol enanthate" OR "Estrogenic Steroids, Alkylated" OR "Estrogens, Conjugated (USP)" OR "estrogens, conjugated synthetic A" OR "estrogens, conjugated synthetic B" OR "Estrogens, Esterified (USP)" OR "Estrone" OR "Ethinyl Estradiol" OR "formononetin" OR "Genistein" OR "glycitein" OR "glycitin" OR "Hexestrol" OR "Infecundin" OR "Mestranol" OR "nylestriol" OR "o,p'-DDT" OR "O-desmethylangolensin" OR "polyestradiol phosphate" OR "Quinestrol" OR "secoisolariciresinol" OR "Zearalenone" OR "Zeranol" OR "2,3-bis(3'-hydroxybenzyl)butane-1,4-diol" OR "2,3-bis(3'-hydroxybenzyl)butyrolactone" OR "4-octylphenol" OR "8-prenylnaringenin" OR "biochanin A" OR "bisphenol A" OR "Chlorotrianisene" OR "Coumestrol" OR "daidzein" OR "Dienestrol" OR "Diethylstilbestrol" OR "diethylstilbestrol dipropionate" OR "Epimestrol" OR "Equol" OR "Estradiol" OR "estradiol enanthate" OR "Estrogenic Steroids, Alkylated" OR "Estrogens, Conjugated (USP)" OR "estrogens, conjugated synthetic A" OR "estrogens, conjugated synthetic B" OR "Estrogens, Esterified (USP)" OR "Estrone" OR "Ethinyl Estradiol" OR "formononetin" OR "Genistein" OR "glycitein" OR "glycitin" OR "Hexestrol" OR "Infecundin" OR "Mestranol" OR "nylestriol" OR "o,p'-DDT" OR "O-desmethylangolensin" OR "polyestradiol phosphate" OR "Quinestrol" OR "secoisolariciresinol" OR "Zearalenone" OR "Zeranol"):ti,ab,kw AND ("Depression" OR "depression" OR "depressive" OR "Mood Disorder" OR "Mood" OR "mood" OR "Cyclothymic Disorder" OR "Dysthymic Disorder" OR "Premenstrual Dysphoric Disorder" OR "Seasonal Affective Disorder" OR "Cyclothymic Disorders" OR "Dysthymic Disorders" OR "Premenstrual Dysphoric Disorders" OR "Seasonal Affective Disorders" OR "Premenstrual Syndrome" OR "premenstrual syndrome" OR "premenstrual symptom*" OR "menstrual symptom*" OR "premenstrual mood" OR "intermenstrual mood" OR "menstrual mood"):ti,ab,kw NOT (("Male" OR "Men" OR "male" OR "males" OR "men") NOT ("Female" OR "Women" OR "Female" OR "Women" OR "girl" OR "girls" OR "girlhood")):ti NOT (("Infant" OR "Child") NOT ("Adolescent" OR "Adult" OR "elderly" OR geriatr*)):ti)

**Search conducted in Emcare**

See Embase

**Search conducted in PsycINFO**

(TX("Estrogens" OR "Estradiol" OR "Estrone" OR "ethinylestradiol" OR "estrogen" OR "estrogen therapy" OR "oral contraceptive agent" OR "hormonal contraception" OR "synthetic estrogen" OR "synthetic estrogens" OR "synthetic oestrogen" OR "synthetic oestrogens" OR "2-Methoxyestradiol" OR "Alkylated Estrogenic Steroids" OR "Catechol Estrogens" OR "Conjugated Estrogens" OR "Equilenin" OR "Equilin" OR "Esterified Estrogens" OR "Estetrol" OR "Estradiol" OR "Estriol" OR "Estrone" OR "Ethinyl Estradiol" OR "Ethinyl Estradiol-Norgestrel Combination" OR "Fulvestrant" OR "Hydroxyestrones" OR "Mestranol" OR "Quinestrol" OR "Catechol Estrogen" OR "Conjugated Estrogen" OR "Esterified Estrogen" OR "Hydroxyestrone" OR "Catechol oestrogen" OR "Conjugated oestrogen" OR "Esterified oestrogen" OR "Catechol oestrogen" OR "Conjugated oestrogen" OR "Esterified oestrogen" OR "Estrogen Replacement" OR "Oestrogen Replacement" OR "Hormone Replacement" OR "Progestin Replacement" OR "Algestone Acetophenide" OR "Algestone Acetophenide" OR "Algestone" OR "Algestone" OR "anordiol" OR "anordiol" OR "anordrin" OR "anordrin" OR "Bisecurin I" OR "Bisecurin I" OR "Centchroman" OR "Centchroman" OR "Chlormadinone Acetate" OR "Chlormadinone Acetate" OR "Cloprostenol" OR "Cloprostenol" OR "CycloProvera" OR "CycloProvera" OR "Cyklabil" OR "Cyklabil" OR "Deposiston" OR "Deposiston" OR "Desogestrel" OR "Desogestrel" OR "diallyl disulfide" OR "diallyl disulfide" OR "diallyl trisulfide" OR "diallyl trisulfide" OR "dienogest" OR "dienogest" OR "Dimethisterone" OR "Dimethisterone" OR "estradiol 17 beta-cypionate" OR "estradiol 17 beta-cypionate" OR "estradiol 3-benzoate" OR "estradiol 3-benzoate" OR "estropipate" OR "estropipate" OR "Ethinyl Estradiol" OR "Ethinyl Estradiol" OR "ethinyl estradiol, levonorgestrel drug combination" OR "ethinyl estradiol, levonorgestrel drug combination" OR "Ethinyl Estradiol-Norgestrel Combination" OR "Ethinyl Estradiol-Norgestrel Combination" OR "Ethynodiol Diacetate" OR "Ethynodiol Diacetate" OR "etonogestrel" OR "etonogestrel" OR "Femovan" OR "Femovan" OR "fluprostenol" OR "fluprostenol" OR "Gestodene" OR "Gestodene" OR "Gestrinone" OR "Gestrinone" OR "gossypol acetic acid" OR "gossypol acetic acid" OR "Infecundin" OR "Infecundin" OR "levonorgestrel butanoate" OR "levonorgestrel butanoate" OR "Levonorgestrel" OR "Levonorgestrel" OR "Lynestrenol" OR "Lynestrenol" OR "Medroxyprogesterone Acetate" OR "Medroxyprogesterone Acetate" OR "Medroxyprogesterone" OR "Medroxyprogesterone" OR "Megestrol" OR "Megestrol" OR "Mestranol" OR "Mestranol" OR "Mifepristone" OR "Mifepristone" OR "neem oil" OR "neem oil" OR "Nonidet P-40" OR "Nonidet P-40" OR "Non-Ovlon" OR "Non-Ovlon" OR "Nonoxynol" OR "Nonoxynol" OR "Noracycline" OR "Noracycline" OR "norelgestromin" OR "norelgestromin" OR "Norethindrone Acetate" OR "Norethindrone Acetate" OR "norethindrone acetate, ethinyl estradiol, ferrous fumarate drug combination" OR "norethindrone acetate, ethinyl estradiol, ferrous fumarate drug combination" OR "norethindrone enanthate" OR "norethindrone enanthate" OR "Norethindrone" OR "Norethindrone" OR "Norethynodrel" OR "Norethynodrel" OR "norgestimate" OR "norgestimate" OR "norgestimate, ethinyl estradiol drug combination" OR "norgestimate, ethinyl estradiol drug combination" OR "Norgestrel" OR "Norgestrel" OR "Norgestrienone" OR "Norgestrienone" OR "Norinyl" OR "Norinyl" OR "NuvaRing" OR "NuvaRing" OR "Octoxynol" OR "Octoxynol" OR "Ortho Evra" OR "Ortho Evra" OR "Oviol" OR "Oviol" OR "SC-11800 EE" OR "SC-11800 EE" OR "ST 1435" OR "ST 1435" OR "sulprostone" OR "sulprostone" OR "trestolone" OR "trestolone" OR "Triptorelin Pamoate" OR "Triptorelin Pamoate" OR "Trisequens" OR "Trisequens" OR "ulipristal acetate" OR "ulipristal acetate" OR "estrogen" OR "estrogens" OR estrogen* OR "oestrogen" OR "oestrogens" OR oestrogen* OR "2,3-bis(3'-hydroxybenzyl)butane-1,4-diol" OR "2,3-bis(3'-hydroxybenzyl)butyrolactone" OR "4-octylphenol" OR "8-prenylnaringenin" OR "biochanin A" OR "bisphenol A" OR "Chlorotrianisene" OR "Coumestrol" OR "daidzein" OR "Dienestrol" OR "Diethylstilbestrol" OR "diethylstilbestrol dipropionate" OR "Epimestrol" OR "Equol" OR "Estradiol" OR "estradiol enanthate" OR "Estrogenic Steroids, Alkylated" OR "Estrogens, Conjugated (USP)" OR "estrogens, conjugated synthetic A" OR "estrogens, conjugated synthetic B" OR "Estrogens, Esterified (USP)" OR "Estrone" OR "Ethinyl Estradiol" OR "formononetin" OR "Genistein" OR "glycitein" OR "glycitin" OR "Hexestrol" OR "Infecundin" OR "Mestranol" OR "nylestriol" OR "o,p'-DDT" OR "O-desmethylangolensin" OR "polyestradiol phosphate" OR "Quinestrol" OR "secoisolariciresinol" OR "Zearalenone" OR "Zeranol" OR "2,3-bis(3'-hydroxybenzyl)butane-1,4-diol" OR "2,3-bis(3'-hydroxybenzyl)butyrolactone" OR "4-octylphenol" OR "8-prenylnaringenin" OR "biochanin A" OR "bisphenol A" OR "Chlorotrianisene" OR "Coumestrol" OR "daidzein" OR "Dienestrol" OR "Diethylstilbestrol" OR "diethylstilbestrol dipropionate" OR "Epimestrol" OR "Equol" OR "Estradiol" OR "estradiol enanthate" OR "Estrogenic Steroids, Alkylated" OR "Estrogens, Conjugated (USP)" OR "estrogens, conjugated synthetic A" OR "estrogens, conjugated synthetic B" OR "Estrogens, Esterified (USP)" OR "Estrone" OR "Ethinyl Estradiol" OR "formononetin" OR "Genistein" OR "glycitein" OR "glycitin" OR "Hexestrol" OR "Infecundin" OR "Mestranol" OR "nylestriol" OR "o,p'-DDT" OR "O-desmethylangolensin" OR "polyestradiol phosphate" OR "Quinestrol" OR "secoisolariciresinol" OR "Zearalenone" OR "Zeranol") AND TX("Depression" OR "depression" OR "depressive" OR "Mood Disorder" OR "Mood" OR "mood" OR "Cyclothymic Disorder" OR "Dysthymic Disorder" OR "Premenstrual Dysphoric Disorder" OR "Seasonal Affective Disorder" OR "Cyclothymic Disorders" OR "Dysthymic Disorders" OR "Premenstrual Dysphoric Disorders" OR "Seasonal Affective Disorders" OR "Premenstrual Syndrome" OR "premenstrual syndrome" OR "premenstrual symptom*" OR "menstrual symptom*" OR "premenstrual mood" OR "intermenstrual mood" OR "menstrual mood") NOT TI(("Male" OR "Men" OR "male" OR "males" OR "men") NOT ("Female" OR "Women" OR "Female" OR "Women""girl" OR "girls" OR "girlhood")) NOT TI(("Infant" OR "Child") NOT ("Adolescent" OR "Adult" OR "elderly" OR geriatr*)) NOT TI("veterinary" OR "rabbit" OR "rabbits" OR "animal" OR "animals" OR "mouse" OR "mice" OR "rodent" OR "rodents" OR "rat" OR "rats" OR "pig" OR "pigs" OR "porcine" OR "horse" OR "horses" OR "equine" OR "cow" OR "cows" OR "bovine" OR "goat" OR "goats" OR "sheep" OR "ovine" OR "canine" OR "dog" OR "dogs" OR "feline" OR "cat" OR "cats") AND (TX("clinical trial" OR "clinical trial" OR "clinical trials" OR "placebo"/ OR placebo* OR placebos* OR "RaCT" OR "RaCTs" OR "randomization" OR random* OR "RCT" OR "RCTs" OR ((single* OR double* OR triple*) NEAR/5 (blind* OR mask*)) OR volunteer*) OR TI("trial" OR "trials")))

### Supplement 2. Quality assessment: GRADE for the effects of hormonal contraceptives on depressive symptoms

Here we describe the GRADE process resulting in the overall judgement of the certainty of evidence of a specific network estimate to be high, moderate, low, or very low. This resulted in an overall judgement per comparison. See **Supplement 2 Supplementary Table 1.** The overall judgement per comparison is based on the judgement of each of GRADE domain: within-study bias, reporting bias, indirectness, imprecision, heterogeneity, and incoherence. The criteria for downgrading the certainty of evidence for a specific comparison within a certain GRADE domain are described below.

##### Supplement 2 Table 1. Overall judgement of the certainty of evidence per network estimate.

| **Formulation** | **Compared to** | **n*** | Within-study bias | Reporting bias | Indirectness | Imprecision | Heterogeneity | Incoherence | **Confidence**  **rating** |
| --- | --- | --- | --- | --- | --- | --- | --- | --- | --- |
| *Mixed evidence* |  |  |  |  |  |  |  |  |  |
| Desogestrel  (75ug) | Ethinylestradiol/desogestrel  (20ug/150ug) | 1 |  |  |  |  |  |  | Very low |
| Desogestrel  (75ug) | Ethinylestradiol/etonogestrel  (15ug/120ug) | 1 |  |  |  |  |  |  | Very low |
| Estradiol/dienogest  (3;2;2;1mg/0;2;3;0mg) | Ethinylestradiol/levonorgestrel  (30ug/150ug) | 1 |  |  |  |  |  |  | Very low |
| Estradiol/nomegestrol  (1.5mg/2.5mg) | Ethinylestradiol/drospirenone  (30ug/3mg) | 1 |  |  |  |  |  |  | Very low |
| Estradiol/nomegestrol  (1.5mg/2.5mg) | Placebo | 1 |  |  |  |  |  |  | Very low |
| Ethinylestradiol/desogestrel  (20ug/150ug) | Ethinylestradiol/etonogestrel  (15ug/120ug) | 1 |  |  |  |  |  |  | Very low |
| Ethinylestradiol/desogestrel  (20ug/150ug) | Ethinylestradiol/devonorgestrel  (20ug/100ug) | 1 |  |  |  |  |  |  | Very low |
| Ethinylestradiol/drospirenone  (30ug/3mg) | Ethinylestradiol/etonogestrel  (15ug/120ug) | 1 |  |  |  |  |  |  | Very low |
| Ethinylestradiol/drospirenone  (30ug/3mg) | Ethinylestradiol/levonorgestrel  (30ug/150ug) | 2 |  |  |  |  |  |  | Very low |
| Ethinylestradiol/levonorgestrel  (20ug/100ug) | Placebo | 1 |  |  |  |  |  |  | Very low |
| Ethinylestradiol/levonorgestrel  (30ug/150ug) | Levonorgestrel  (30ug) | 2 |  |  |  |  |  |  | Very low |
| Ethinylestradiol/levonorgestrel  (30ug/150ug) | Placebo | 4 |  |  |  |  |  |  | Very low |
| Levonorgestrel  (30ug) | Placebo | 2 |  |  |  |  |  |  | Very low |
|  |  |  |  |  |  |  |  |  |  |
| *Indirect evidence* |  |  |  |  |  |  |  |  |  |
| Desogestrel  (75ug) | Estradiol/dienogest  (3;2;2;1mg/ 0;2;3;0mg) | - |  |  |  |  |  |  | Very low |
| Desogestrel  (75ug) | Estradiol/nomegestrol  (1.5mg/2.5mg) | - |  |  |  |  |  |  | Very low |
| Desogestrel  (75ug) | Ethinylestradiol/drospirenone  (30ug/3ug) | - |  |  |  |  |  |  | Very low |
| Desogestrel  (75ug) | Ethinylestradiol/levonorgestrel  (20ug/100ug) | - |  |  |  |  |  |  | Very low |
| Desogestrel  (75ug) | Ethinylestradiol/levonorgestrel  (30ug/150ug) | - |  |  |  |  |  |  | Very low |
| Desogestrel  (75ug) | Levonorgestrel  (30ug) | - |  |  |  |  |  |  | Very low |
| Desogestrel  (75ug) | Placebo | - |  |  |  |  |  |  | Very low |
| Estradiol/dienogest  (3;2;2;1mg/ 0;2;3;0mg) | Estradiol/nomegestrol  (1.5mg/2.5mg) | - |  |  |  |  |  |  | Very low |
| Estradiol/dienogest  (3;2;2;1mg/ 0;2;3;0mg) | Ethinylestradiol/desogestrel  (20ug/150ug) | - |  |  |  |  |  |  | Very low |
| Estradiol/dienogest  (3;2;2;1mg/ 0;2;3;0mg) | Ethinylestradiol/drospirenone  (30ug/3mg) | - |  |  |  |  |  |  | Very low |
| Estradiol/dienogest  (3;2;2;1mg/ 0;2;3;0mg) | Ethinylestradiol/etonogestrel  (15ug/120ug) | - |  |  |  |  |  |  | Very low |
| Estradiol/dienogest  (3;2;2;1mg/ 0;2;3;0mg) | Ethinylestradiol/levonorgestrel  (20ug/100ug) | - |  |  |  |  |  |  | Very low |
| Estradiol/dienogest  (3;2;2;1mg/ 0;2;3;0mg) | Levonorgestrel  (30ug) | - |  |  |  |  |  |  | Very low |
| Estradiol/dienogest  (3;2;2;1mg/ 0;2;3;0mg) | Placebo | - |  |  |  |  |  |  | Very low |
| Estradiol/nomegestrol  (1.5mg/2.5mg) | Ethinylestradiol/desogestrel  (20ug/150ug) | - |  |  |  |  |  |  | Very low |
| Estradiol/nomegestrol  (1.5mg/2.5mg) | Ethinylestradiol/etonogestrel  (15ug/120ug) | - |  |  |  |  |  |  | Very low |
| Estradiol/nomegestrol  (1.5mg/2.5mg) | Ethinylestradiol/levonorgestrel  (20ug/100ug) | - |  |  |  |  |  |  | Very low |
| Estradiol/nomegestrol  (1.5mg/2.5mg) | Ethinylestradiol/levonorgestrel  (30ug/150ug) | - |  |  |  |  |  |  | Very low |
| Estradiol/nomegestrol  (1.5mg/2.5mg) | Levonorgestrel  (30ug) | - |  |  |  |  |  |  | Very low |
| Ethinylestradiol/desogestrel  (20ug/150ug) | Ethinylestradiol/drospirenone  (30ug/3mg) | - |  |  |  |  |  |  | Very low |
| Ethinylestradiol/desogestrel  (20ug/150ug) | Ethinylestradiol/levonorgestrel  (30ug/150ug) | - |  |  |  |  |  |  | Very low |
| Ethinylestradiol/desogestrel  (20ug/150ug) | Levonorgestrel  (30ug) | - |  |  |  |  |  |  | Very low |
| Ethinylestradiol/desogestrel  (20ug/150ug) | Placebo | - |  |  |  |  |  |  | Very low |
| Ethinylestradiol/drospirenone  (30ug/3mg) | Ethinylestradiol/levonorgestrel  (20ug/100ug) | - |  |  |  |  |  |  | Very low |
| Ethinylestradiol/drospirenone  (30ug/3mg) | Levonorgestrel  (30ug) | - |  |  |  |  |  |  | Very low |
| Ethinylestradiol/drospirenone  (30ug/3mg) | Placebo | - |  |  |  |  |  |  | Very low |
| Ethinylestradiol/etonogestrel  (15ug/120ug) | Ethinylestradiol/levonorgestrel  (20ug/100ug) | - |  |  |  |  |  |  | Very low |
| Ethinylestradiol/etonogestrel  (15ug/120ug) | Ethinylestradiol/levonorgestrel  (30ug/150ug) | - |  |  |  |  |  |  | Very low |
| Ethinylestradiol/etonogestrel  (15ug/120ug) | Levonorgestrel  (30ug) | - |  |  |  |  |  |  | Very low |
| Ethinylestradiol/etonogestrel  (15ug/120ug) | Placebo | - |  |  |  |  |  |  | Very low |
| Ethinylestradiol/levonorgestrel  (20ug/100ug) | Ethinylestradiol/levonorgestrel  (30ug/150ug) | - |  |  |  |  |  |  | Very low |
| Ethinylestradiol/levonorgestrel  (20ug/100ug) | Levonorgestrel  (30ug) | - |  |  |  |  |  |  | Very low |
| no concerns some concerns high concerns | | | | | | | | | |

*Refers to the number of studies investigating the comparison.

The table describes the judgement of certainty of evidence for each of the GRADE domains that results in an overall judgement of certainty of evidence per comparison. Ethinylestradiol/dienogest (3;2;2;1mg/0;2;3;0mg) is a multiphasic combined oral contraceptive that has four different dosages of hormones throughout a 4-week cycle. The dosages before the / refer to the different dosages of ethinylestradiol, and the ones after the / to those of dienogest.

#### Within study bias

We rated the overall risk of bias within each study according to the rating of the domain rated with the highest risk of bias. Of the trials included in the meta-analysis, we judged six trials to have a high risk of bias,(1-6) five trials to have some concerns,(7-11) and only one study to have low risk of bias.(12) Most often the bias arose from missing outcome data.(1-6, 8, 11, 13) In these cases, plus two other trials, there were also some(1-3, 5, 7-11, 13) or high(4, 6, 14) concerns about the selection of the reported results. Two studies that were not eligible for inclusion in the network meta-analysis as the interventions were not connected with the network (13, 14) were also judged to have a high risk of bias due to missing data on the outcome(13) or due to selection of the reported results.(14) See also **Supplement 7 Table 2**.

##### Supplement 2 Table 2. Study specific risk of bias judgements

| **Study** | Randomization | Deviation from intended intervention | Missing outcome data | Measurements of outcome | Selection of the reported result | **Overall** |
| --- | --- | --- | --- | --- | --- | --- |
| *Included in the network meta-analysis* |  |  |  |  |  |  |
| Battaglia |  |  |  |  |  |  |
| Davis |  |  |  |  |  |  |
| Elaut |  |  |  |  |  |  |
| Engman |  |  |  |  |  |  |
| Graham |  |  |  |  |  |  |
| Kelly |  |  |  |  |  |  |
| Lundin |  |  |  |  |  |  |
| O'Connell |  |  |  |  |  |  |
| Sangthawan |  |  |  |  |  |  |
| Winkler |  |  |  |  |  |  |
| Witjes |  |  |  |  |  |  |
| Zethraeus |  |  |  |  |  |  |
|  |  |  |  |  |  |  |
| *Included in the systematic review only* |  |  |  |  |  |  |
| Greco |  |  |  |  |  |  |
| Legro |  |  |  |  |  |  |
| low concerns, some concerns, high concerns | | | |  |  |  |

The table shows the judged risk of bias in each domain for every study included in the review. Risk of bias were determined using the Cochrane Risk of Bias tool.(15)

We downgraded the confidence rating for a network estimate by one level when the contributions were on average coming from “some concerns” risk of bias comparisons or with two levels when the contributions were mostly coming from “high” risk of bias comparisons.(16) See **Supplement 2 Figure** **1**.

##### Supplement 2 Figure 1. Network estimate specific risk of bias judgements

RoB judgement for average quality of network estimate. Each bar represents a relative treatment effect estimated from the network shown in **Figure 2**. White vertical lines separate the percentage contribution of different studies. Each bar shows the percentage contribution from studies judged to be at no concerns, some concerns, and high concerns. See the text above below the corresponding header “within-study bias” for details about rules used to judge comparison as having no, some or high concerns. Ethinylestradiol/dienogest (3;2;2;1mg/0;2;3;0mg) is a multiphasic combined oral contraceptive that has four different dosages of hormones throughout a 4-week cycle. The dosages before the / refer to the different dosages of ethinylestradiol, and the ones after the / to those of dienogest.

#### Reporting bias

The symmetrical comparison adjusted funnel plot did not suggest presence of overall publication bias in comparison with placebo. See **Supplement 2 Figure 2**. We also managed to retrieve additional supplementary and unpublished data that showed similar effect sizes as the published data. Hence, none of the studies was downgraded for potential publication bias.(16)

##### Supplement 2 Figure 2. Funnel plot of randomized clinical trials of hormonal contraceptives versus placebo

The color of the nodes corresponds with the type of hormonal contraceptives compared in the specific study (arm) (combined oral contraceptives; progesterone-only pill; vaginal ring; or placebo).

#### Indirectness

The distribution of age was balanced across comparisons. See **Table 1.** However, full assessment of transivity was challenging as information on many potential effect moderators, such as previously negative experiences with hormonal contraceptive use and previous MDD, was not provided. Moreover, a major group of users, first-time users, was not or only very limited at most, included in the trials. Hence, the sample was not fully representative of hormonal contraceptive users. Also, the majority of interventions were only poorly connected to the network and therefore the evaluation of transitivity for these comparisons is unclear.(16) Hence, we downgraded comparisons to "some concerns" if they did not include first-time users or a group of women with previously negative experiences with hormonal contraceptive use, and additionally downgraded to "major concerns" if the comparison was only investigated once for this domain. Subsequently, we downgraded the confidence rating for a network estimate by one level when the contributions were on average coming from “some concerns” risk of bias comparisons or with two levels when the contributions were predominantly coming from “high” risk of bias comparisons.(16) See **Supplement 2 Figure 3.**

##### Supplement 2 Figure 3. Network estimate specific indirectness

Indirectness judgement for average quality of network estimate. Each bar represents a relative treatment effect estimated from the network shown in **Figure 2**. White vertical lines separate the percentage contribution of different studies. Each bar shows the percentage contribution from studies judged to be at no concerns, some concerns, and high concerns. See the text above below the corresponding header “indirectness” for details about rules used to judge comparison as having no, some or high concerns. Ethinylestradiol/dienogest (3;2;2;1mg/0;2;3;0mg) is a multiphasic combined oral contraceptive that has four different dosages of hormones throughout a 4-week cycle. The dosages before the / refer to the different dosages of ethinylestradiol, and the ones after the / to those of dienogest.

#### Imprecision

We considered 0.20 as the minimally clinically meaningful important difference. This was based on the rule of thumb for small effect sizes.(17) A rating of ‘major concerns’ was assigned to comparisons if the 95% credible interval of the effect extended beyond the area of equivalence on the opposite side of the no effect line as the point estimate (credible interval ranged from below -0.20 to beyond 0.20), so that the estimated treatment effect is compatible with clinically important effects in both directions. A rating of ‘some concerns’ was assigned if the credible interval extended into but not beyond the area of equivalence on the opposite side of the no effect line. 'No concerns’ were assigned if the credible interval was entirely on one side of the no effect line (below -0.20, or beyond 0.20), or if it was entirely within the area of equivalence (beyond -0.20 and below 0.20).(16)

#### Heterogeneity

Imprecision and incoherence are related to heterogeneity. Hence, we only rated heterogeneity for comparisons that were not already downgraded on the domains of imprecision and incoherence. In these two cases, the amount of heterogeneity was assessed with the I^2^. The I^2^ of the network meta-analysis was acceptable (43.7%), suggesting moderate between-trial heterogeneity. Hence, we rated these network estimate as “some concerns” for heterogeneity.

#### Incoherence

For incoherence, we looked at the results of the node splitting and we downgraded the comparisons with important inconsistency (p<0.10).(16) For the majority of closed loops (five out of eight) the indirect and direct effects were in the same direction. For a minority of closed loops however (i.e., ethinylestradiol/drospirenone [30ug/3mg] versus estradiol/nomegestrel [1.5mg/2.5mg], placebo versus estradiol/nomegestrel [1.5mg/2.5mg], and ethinylestradiol/levonorgestrel [30ur/150ug] versus ethinylestradiol/drospirenone [30ug/3mg]) these effects were in the opposite direction, though these differences were not significant (p = 0.17, p = 0.17, and p = 0.19, respectively). As none of the p-values were below 0.10, we did not downgrade any of these comparisons. See **Supplement 2 Figure 4**. In case there was only direct or indirect evidence for a comparison, we rated the certainty of evidence for the incoherence domain as "some concerns".(16) See **Supplement 2 Table 1**.

##### Supplement 2 Figure 4. Estimates of direct and indirect effects

Summary of a node‐split analysis consisting of nine separate node‐splitting models that show direct, indirect and the network estimate.

### References

1. Kelly S, Davies E, Fearns S, McKinnon C, Carter R, Gerlinger C, et al. Effects of oral contraceptives containing ethinylestradiol with either drospirenone or levonorgestrel on various parameters associated with well-being in healthy women: a randomized, single-blind, parallel-group, multicentre study. Clin Drug Investig. 2010;30(5):325-36.

2. Lundin C, Danielsson KG, Bixo M, Moby L, Bengtsdotter H, Jawad I, et al. Combined oral contraceptive use is associated with both improvement and worsening of mood in the different phases of the treatment cycle-A double-blind, placebo-controlled randomized trial. Psychoneuroendocrinology. 2017;76:135-43.

3. Witjes H, Creinin MD, Sundström-Poromaa I, Martin Nguyen A, Korver T. Comparative analysis of the effects of nomegestrol acetate/17 β-estradiol and drospirenone/ethinylestradiol on premenstrual and menstrual symptoms and dysmenorrhea. Eur J Contracept Reprod Health Care. 2015;20(4):296-307.

4. Battaglia C, Morotti E, Persico N, Battaglia B, Busacchi P, Casadio P, et al. Clitoral vascularization and sexual behavior in young patients treated with drospirenone-ethinyl estradiol or contraceptive vaginal ring: a prospective, randomized, pilot study. J Sex Med. 2014;11(2):471-80.

5. Engman J, Sundström Poromaa I, Moby L, Wikström J, Fredrikson M, Gingnell M. Hormonal Cycle and Contraceptive Effects on Amygdala and Salience Resting-State Networks in Women with Previous Affective Side Effects on the Pill. Neuropsychopharmacology. 2018;43(3):555-63.

6. Winkler UH, Ferguson H, Mulders JA. Cycle control, quality of life and acne with two low-dose oral contraceptives containing 20 microg ethinylestradiol. Contraception. 2004;69(6):469-76.

7. Graham CA, Ramos R, Bancroft J, Maglaya C, Farley TM. The effects of steroidal contraceptives on the well-being and sexuality of women: a double-blind, placebo-controlled, two-centre study of combined and progestogen-only methods. Contraception. 1995;52(6):363-9.

8. O'Connell K, Davis AR, Kerns J. Oral contraceptives: side effects and depression in adolescent girls. Contraception. 2007;75(4):299-304.

9. Sangthawan M, Taneepanichskul S. A comparative study of monophasic oral contraceptives containing either drospirenone 3 mg or levonorgestrel 150 microg on premenstrual symptoms. Contraception. 2005;71(1):1-7.

10. Elaut E, Buysse A, De Sutter P, De Cuypere G, Gerris J, Deschepper E, et al. Relation of androgen receptor sensitivity and mood to sexual desire in hormonal contraception users. Contraception. 2012;85(5):470-9.

11. Davis SR, Bitzer J, Giraldi A, Palacios S, Parke S, Serrani M, et al. Change to either a nonandrogenic or androgenic progestin-containing oral contraceptive preparation is associated with improved sexual function in women with oral contraceptive-associated sexual dysfunction. J Sex Med. 2013;10(12):3069-79.

12. Zethraeus N, Dreber A, Ranehill E, Blomberg L, Labrie F, von Schoultz B, et al. A first-choice combined oral contraceptive influences general well-being in healthy women: a double-blind, randomized, placebo-controlled trial. Fertil Steril. 2017;107(5):1238-45.

13. Greco T, Graham CA, Bancroft J, Tanner A, Doll HA. The effects of oral contraceptives on androgen levels and their relevance to premenstrual mood and sexual interest: a comparison of two triphasic formulations containing norgestimate and either 35 or 25 microg of ethinyl estradiol. Contraception. 2007;76(1):8-17.

14. Legro RS, Pauli JG, Kunselman AR, Meadows JW, Kesner JS, Zaino RJ, et al. Effects of continuous versus cyclical oral contraception: a randomized controlled trial. J Clin Endocrinol Metab. 2008;93(2):420-9.

15. Sterne JAC, Savović J, Page MJ, Elbers RG, Blencowe NS, Boutron I, et al. RoB 2: a revised tool for assessing risk of bias in randomised trials. BMJ. 2019;366:l4898.

16. Nikolakopoulou A, Higgins JPT, Papakonstantinou T, Chaimani A, Del Giovane C, Egger M, et al. CINeMA: An approach for assessing confidence in the results of a network meta-analysis. PLoS Med. 2020;17(4):e1003082.

17. Cohen J. Statistical power analysis for the behavioral sciences. Revised edition ed. London: Academic Press; 1977.
